# Novel Dual 5-HT_7_ Antagonists and Sodium Channel Inhibitors as Potential Therapeutic Agents with Antidepressant and Anxiolytic Activities

**DOI:** 10.3390/ph18101485

**Published:** 2025-10-02

**Authors:** Anna Czopek, Paulina Koczurkiewicz-Adamczyk, Katarzyna Wójcik-Pszczoła, Daria Kornas, Wojciech Sitko, Adam Bucki, Michał Sapa, Krzysztof Kamiński, Grzegorz Satała, Beata Duszyńska, Andrzej J. Bojarski, Gniewomir Latacz, Jacek Czopek, Joanna Szpor, Pola Dryja, Kinga Sałat

**Affiliations:** 1 Department of Medicinal Chemistry, Faculty of Pharmacy, Jagiellonian University Medical College, 9 Medyczna St., 30-688 Kraków, Poland; 2 Department of Pharmaceutical Biochemistry, Faculty of Pharmacy, Jagiellonian University Medical College, 9 Medyczna St., 30-688 Krakow, Poland; 3 Department of Medicinal Chemistry, Maj Institute of Pharmacology, Polish Academy of Sciences, 12 Smętna St., 31-343 Kraków, Poland; 4 Department of Phytochemistry, Maj Institute of Pharmacology, Polish Academy of Sciences, 12 Smętna St., 31-343 Kraków, Poland; 5 Department of Technology and Biotechnology of Drugs, Faculty of Pharmacy, Jagiellonian University Medical College, 9 Medyczna St., 30-688 Kraków, Poland; 6 Department of Pathomorphology, Jagiellonian University Medical College, 16 Grzegórzecka St., 31-008 Kraków, Poland; 7 Department of Pharmacodynamics, Chair of Pharmacodynamics, Faculty of Pharmacy, Jagiellonian University Medical College, 9 Medyczna St., 30-688 Kraków, Poland

**Keywords:** hydantoin, 1-(2-biphenyl)piperazine, 5-HT_7_ ligand, sodium channel inhibitor, antidepressant-like activity, anxiolytic-like activity

## Abstract

**Background/Objectives:** The study aimed to pharmacologically evaluate dually acting ligands, 5-HT_7_ antagonists and sodium channel inhibitors, as potential therapeutic agents for the treatment of depression, anxiety, and neuropathic pain. The designed dual ligands combined structural fragments of LP-12 (a 5-HT_7_ receptor ligand) and phenytoin (a sodium channel blocker). **Methods:** A series of 1-(2-biphenyl)piperazine derivatives with a hydantoin core was synthesized and evaluated for 5-HT_7_ receptor affinity and sodium channel inhibition. The most potent ligands were further analyzed using molecular docking, cytotoxicity assays (MTT, LDH), and in vitro metabolism studies, including microsomal stability and CYP450 inhibition. In vivo pharmacological effects were assessed in behavioral models: forced swim test, four-plate test, and a streptozotocin (STZ)-induced diabetic neuropathy model in mice. **Results:** Compounds **10** and **20** exhibited high 5-HT_7_ receptor affinity (K_i_ < 10 nM) and potent sodium channel inhibition (>80% at 1 µM). Docking studies revealed binding modes consistent with established 5-HT_7_ ligands. Compound **10** showed lower cytotoxicity than compound **20** in both HepG2 and SH-SY5Y cells and was therefore selected for further evaluation. Metabolic profiling indicated improved microsomal stability relative to verapamil and a low risk of CYP-mediated drug–drug interactions. In vivo, compound **10** produced significant antidepressant- and anxiolytic-like effects, though it failed to reduce neuropathic pain symptoms in the STZ-induced model. **Conclusions:** Compound **10** shows potential for mood disorder treatment, but further refinement may be needed to improve analgesic efficacy.

## 1. Introduction

Pain and mood disturbances, particularly depression and anxiety, frequently co-occur and exacerbate one another, creating a bidirectional relationship mediated by overlapping neurobiological pathways, including dysregulation of the hypothalamic–pituitary–adrenal (HPA) axis, neuroinflammatory responses, and alterations in monoaminergic neurotransmission [1,2]. As such, co-occurring and sharing overlapping neurobiological mechanisms, pain and depression management presents a substantial clinical challenge [3].

The serotonin 5-HT_7_R is widely expressed in the central nervous system (e.g., thalamus, hypothalamus, hippocampus, cortex, and spinal cord) and has been implicated in modulating pain, mood, and neuroplasticity. Notably, 5-HT_7_Rs in the spinal cord and pain pathways appear to play a key role in nociceptive processing [4]. Selective 5-HT_7_ agonists have shown analgesic effects in animal models. For instance, preclinical studies have demonstrated that selective 5-HT_7_ receptor agonists, such as LP-211 or LP-12, can modulate pain signaling pathways at various levels of the central nervous system (CNS) and reduce hyperalgesia and allodynia in rodent pain assays. In the formalin test, compound LP-211 dose-dependently decreased pain behaviors in mice [5,6,7]. In addition to pain modulation, the 5-HT_7_ receptor has been implicated in, among others, mood regulation, circadian rhythm, and cognitive function. Interestingly, the influence of the 5-HT_7_ receptor on depression-like behaviors appears to be complex and inconsistent across studies [8,9]. While some evidence points to antidepressant and anxiolytic effects resulting from receptor blockade [10], other findings suggest that receptor activation may exert a similar influence [9]. Although direct 5-HT_7_ agonists are less studied in depression models, the procognitive and neuroplasticity-promoting effects of 5-HT_7_ agonism suggest a potential to positively influence mood and recovery from stress. Notably, two selective agonists (LP-211 and LP-12) have been used as research tools to probe 5-HT_7_-mediated effects on neuronal morphology and function, and their pharmacology provides a template for developing novel CNS-active agents. LP-12 displayed a high-affinity for 5-HT_7_R (K_i_ = 0.13 nM) with excellent selectivity over 5-HT_1A_, 5-HT_2A_, and D_2_ receptors [11].

Voltage-gated sodium channel blockers are well known for their anticonvulsant and analgesic effects, including efficacy in neuropathic pain [12], and some also show benefits in mood disorders [13,14]. Lamotrigine, for example, is approved for bipolar disorder and works as an antidepressant drug by stabilizing neuronal activity through sodium channel inhibition [15]. It reduces depressive episodes without triggering mania and shows antidepressant-like effects in animal models [16]. Phenytoin (5,5-diphenylhydantoin) is an anticonvulsant that blocks sodium channels and has long been used off-label for neuropathic pain conditions such as trigeminal neuralgia [17]. Its ability to reduce neural excitability and inflammation may also contribute to reported benefits in psychiatric symptoms, including anxiety and aggression [18,19].

Previously synthesized hydantoin derivatives with various substitutions have demonstrated a broad pharmacological profile, including anticonvulsant, analgesic, antiarrhythmic, anticancer, and antipsychotic activities [17,20,21,22,23,24,25]. These findings demonstrate that structural modifications within the hydantoin core can lead to diverse biological activities.

**Figure 1:**
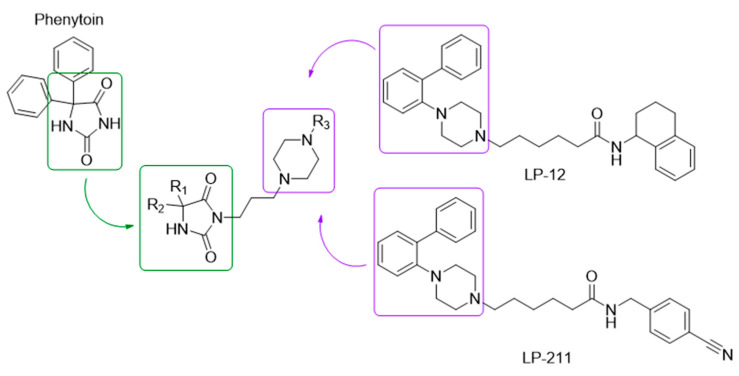
The general structure of the designed compounds is based on phenytoin (sodium channel blocker, outlined with a green line) and LP-12, LP-211 (selective 5-HT_7_ receptor agonists, outlined with a purple line).

In this study, we designed molecules containing structural fragments of known 5-HT_7_R agonists and sodium channel blockers, such as phenytoin (Figure 1). The design was based on LP-12 and LP-211 scaffolds, 5-HT_7_ agonists characterized by a long-chain arylpiperazine structure. In our novel compounds, we incorporated the 1-(2-biphenyl)piperazine fragment typical of 5-HT_7_R agonists but replaced the amide linker of LP-12 with a hydantoin ring from phenytoin. This substitution may preserve the receptor-binding conformation at 5-HT_7_ while also introducing sodium channel blocking activity. Such dual-acting compounds can modulate neuropathic pain pathways and help regulate emotional processing in mood-related disorders.

The new compounds differ in three key structural features: the substituents on the hydantoin ring, the length of the alkyl linker connecting the hydantoin and piperazine moieties, and the substituents on the 1-(2-biphenyl)piperazine fragment. These modifications were designed to assess their impact on binding affinity, safety, and stability.

Therefore, in this study, we designed and synthesized novel compounds combining structural features of LP-12 and phenytoin to achieve dual activity as 5-HT_7_R agonists and sodium channel blockers. The compounds were evaluated for their affinity towards the 5-HT_7_R, and the most active derivatives were further tested for sodium channel inhibition. Given the known toxicity of phenytoin, the final compound’s cytotoxicity was assessed using MTT and LDH (lactate dehydrogenase) assays. Molecular modeling was also conducted to investigate binding poses within the 5-HT_7_R. Additionally, metabolic stability in human liver microsomes and potential drug–drug interactions (DDIs) were evaluated. Finally, the biological (in vivo) activity of the selected compound was assessed in a streptozotocin (STZ)-induced diabetic neuropathy mouse model, as well as in behavioral tests for antidepressant-like (forced swim test (FST)) and anxiolytic-like (four-plate test, (FPT)) effects.

## 2. Results and Discussion

### 2.1. Chemistry

**Scheme 1:**
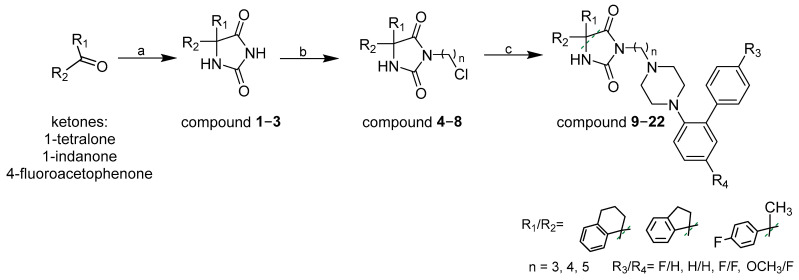
Synthetic routes for the final compounds **9**–**22**. Reagents and conditions: (a) KCN, (NH_4_)_2_CO_3_, 50% ethyl alcohol, 16 h, 56–60 °C; (b) appropriate dihalogenoalkanes, K_2_CO_3_, acetone, reflux 7–20 h; (c) appropriate 1-(2-biphenyl)piperazine, K_2_CO_3_, MeCN, 100 °C, 25 W (MW), 45 min. The starting hydantoins as well as the intermediates (**1**–**8**) have already been reported in [20,21,22,23]. Ketones used for the synthesis were commercially available.

New 1-(2-biphenyl)piperazine derivatives (**9**–**22**) were synthesized in moderate to good yields (44–79%), as shown in Scheme 1. The initial imidazolidine-2,4-diones were prepared from the corresponding ketones via the Bucherer–Berg reaction [26]. Intermediate compounds (**4**–**8**) were obtained by alkylation of appropriate imidazolidine-2,4-dione derivatives at the N3 position using dihalogenoalkanes, followed by coupling with 1-(2-biphenyl)piperazine derivatives (**I**–**IV**). Thin-layer chromatography (TLC) was employed to monitor reaction progress. The final compounds (**9**–**22**) were characterized and their purity established by NMR, LC/MS, and HPLC. Complete analytical and physical data are included in the materials and methods section.

**Scheme 2:**
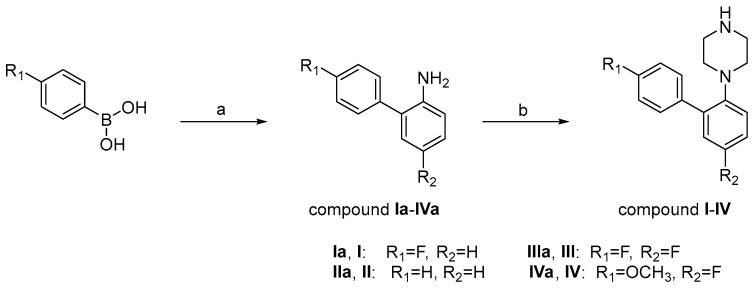
Reagents and conditions for the preparation of 1-(2-biphenyl)piperazine derivatives: (a) appropriate amine, Pd(dppf)Cl_2_, K_2_CO_3_, toluene, water, 100 °C, 24 h; (b) *bis*(2-chloroethyl)amine hydrochloride, monomethyl ether of ethylene glycol, 150 °C, 24 h. Piperazine **I**, **II**, and **IV** have been previously described [28,29]. Appropriate boronic acids and the intermediate [1,1′-biphenyl]-2-amine (**IIa**) were commercially available.

The 1-(2-biphenyl)piperazine derivatives were synthesized in accordance with previously reported procedures (Scheme 2). In the first step, the corresponding intermediates were obtained via the Suzuki–Miyaura cross-coupling reaction [27]. Subsequently, the [1,1′-biphenyl]-2-amines (**Ia**–**IVa**) underwent cyclization with *bis*(2-chloroethyl)amine hydrochloride to yield the desired heterocyclic piperazine derivatives (**I**–**IV**).

### 2.2. In Vitro Binding Affinity and Functional Profile for 5-HT_7_R and Sodium Channels

As part of preliminary studies, three novel compounds (**9**, **10**, and **11**; Table 1) were synthesized, with a spirohydantoin moiety fused with a tetralin ring and connected to a 1-(2-biphenyl)piperazine fragment via alkyl linkers of different lengths: pentylene (compound **9**), butylene (compound **10**), and propylene (compound **11**). These compounds were first evaluated for their binding affinity to the 5-HT_7_R and showed high affinity, with K_i_ values < 50 nM (6 ± 1 nM, 8 ± 2 nM, and 46 ± 11 nM, respectively). The compounds were then evaluated for their inhibitory activity at sodium channels (site 2). In contrast with the relatively similar 5-HT_7_R binding affinity, the assay revealed greater variation in sodium channel inhibition. At a concentration of 100 µM, compound **10** (with butylene linker) exhibited the strongest inhibition (107.2%), while compound **11** (with propylene linker) showed weaker activity (83.6%), and compound **9** (with pentylene linker) demonstrated the lowest inhibition (55.6%). Based on the aforementioned data indicating the highest affinity toward 5-HT_7_R and sodium channels, the butylene linker was chosen for the synthesis of a series of compounds with varying substituents at the 5-position of the hydantoin ring and within the 1-(2-biphenyl)piperazine fragment.

Based on the binding data (Table 1), all compounds containing a butylene linker (**10** and **12**–**22**) exhibited high to moderate affinity for the 5-HT_7_R (K_i_ = 5–143 nM). The highest affinity was observed for compound **20** (K_i_ = 5 nM), which features 4-fluorophenyl and methyl substituents at 5-position of the hydantoin ring and an unsubstituted 1-(2-biphenyl)piperazine moiety (H/H). In general, derivatives bearing a spirohydantoin scaffold and either no substituents (H/H) or a single fluorine atom at the 4′-position (F/H) on the 1-(2-biphenyl)piperazine fragment (compounds **10**, **12**, **15**, and **16**) demonstrated high affinity toward the 5-HT_7_R (K_i_ < 20 nM). In contrast, the introduction of two substituents (e.g., F/F or F/OCH_3_) on the 1-(2-biphenyl)piperazine moiety (compounds **13**, **14**, **17**, **18**, **21**, **22**) led to a significant reduction in receptor binding affinity. Compounds with an indene moiety at the 5-position of the hydantoin scaffold (**15**–**18**) demonstrated superior affinity compared to those bearing either a tetralin group or 4-fluorophenyl and methyl substituents at the same position of the scaffold.

For the most active 5-HT_7_R ligands, compounds **10** and **20**, which exhibited K_i_ values below 10 nM, the inhibitory activity toward sodium channels (site 2) was also evaluated (Table 1). The obtained results revealed very strong and comparable inhibitory activity for both compounds, with compound **10** inhibiting 99.3% and 88.6%, and compound **20** inhibiting 97.8% and 83.0% of specific binding at 10 µM and 1 µM, respectively.

Compounds **10** and **20** demonstrated the most favorable activity profile, combining high affinity for the 5-HT_7_R receptor (Ki < 10 nM) with potent sodium channel inhibition (>80% at 1 µM). This effect may be due to the presence of a butylene linker, which provides optimal spatial orientation of the pharmacophores, and (un)substitutions in the 1-(2-biphenyl)piperazine moiety (compound **10**: F/H or compound **20**: H/H). These structural features appear to facilitate both efficient receptor binding and sodium channel inhibition, underscoring their importance in achieving a balanced dual activity profile.

**Figure 2:**
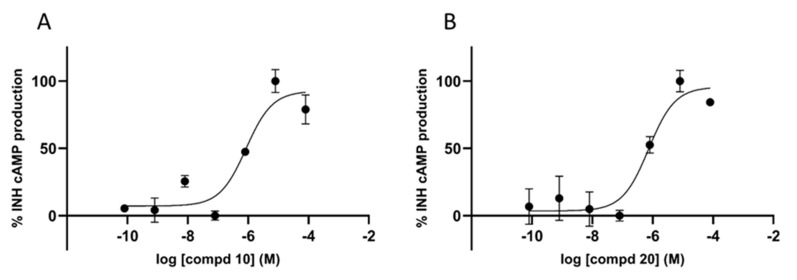
Functional activity profile of compounds **10** (**A**) and **20** (**B**) at 5-HT_7_R.

Additionally, compounds **10** and **20** were assessed in a functional cAMP assay using HEK-293 cells overexpressing the human 5--HT_7_R, allowing the determination of their functional pharmacological profiles. The data showed that both compounds act as antagonists, with K_b_ values of 73 nM and 72 nM, respectively (Figure 2), indicating high antagonist potency. No agonistic activity was observed across the tested concentration range (EC_50_ not reached), confirming their antagonist profile at 5-HT_7_R.

### 2.3. Molecular Modeling Studies

Both ligands, **10** and **20**, were successfully docked into the orthosteric binding site of the 5-HT_7_ receptor model. Among the evaluated stereoisomers, the R-enantiomer of **10** and the S-enantiomer of **20** exhibited the most favorable binding conformations, based on docking scores and interaction profiles.

The ligands adopted highly similar binding poses, reflecting their pharmacological profiles. A crucial electrostatic interaction was observed between the protonated nitrogen atom of the piperazine moiety and the carboxylate side chain of Asp3.32, a well-characterized anchoring interaction in class A GPCR ligand binding [31]. The biphenyl scaffold was positioned within the orthosteric pocket, engaging in π–π stacking interactions with aromatic residues Phe6.51 and Phe6.52, contributing significantly to ligand stabilization.

**Figure 3:**
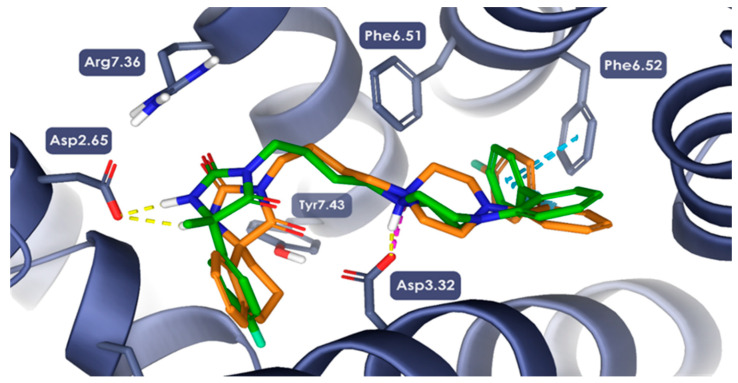
Predicted binding modes of **10** (orange) and **20** (green) within the 5-HT_7_ receptor model, presented from the extracellular side. For clarity, the extracellular loop 2 (ECL2) has been hidden. Both ligands adopt highly similar orientations within the binding site, with the key ionic interaction—a salt bridge between the protonated piperazine nitrogen atom and Asp3.32—highlighted by a purple dashed line. Within the orthosteric pocket, the biphenyl moiety forms π–π stacking with Phe6.52 (azure dashed line). The phenylhydantoin (**20**) and spiro-hydantoin (**10**) fragments occupy an additional subpocket between transmembrane helices TM2 and TM3 and are further stabilized via hydrogen bonding with Asp2.65 (yellow dashed line).

Additional moieties of both ligands—the spiro-substituted dihydronaphthalene ring of **10** and the 4-fluorophenyl moiety of **20**—extended towards an allosteric hydrophobic subpocket formed between transmembrane helices TM2 and TM3. These regions were stabilized by polar interactions involving the hydantoin motif. Specifically, the nitrogen atom of the hydantoin ring formed a hydrogen bond with the side chain of Asp2.65. Moreover, the adjacent carbonyl group was oriented towards Arg7.36, a residue previously implicated in ligand-selective recognition in the 5-HT_7_R, thereby enabling potential polar interactions within a dynamic system (Figure 3) [31,32,33].

### 2.4. In Vitro Evaluation of Hepato- and Neurotoxicity Activity

**Figure 4:**
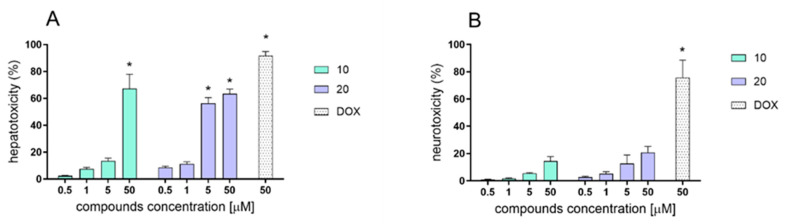
Cytotoxic effect of compound **10** and **20** in HepG2 and SHSY-5Y cells. Cells were seeded at a density of 5 × 10^3^ cells/well and after overnight culture exposed to growing concentrations (0.5–100 µM) of tested compounds for 24 h. Hepatocytotoxicity (**A**) and neurotoxicity (**B**) were measured by CyQUANT™ LDH Cytotoxicity Assay Kit (Waltham, MA, USA), assessing LDH release into the culture medium as an indicator of plasma membrane integrity. Bars represent mean ± SEM of percent cytotoxicity obtained in three independent experiments. Results were compared to positive control—doxorubicin (DOX). * Statistical significance versus control (*p* < 0.05, Wilcoxon signed-rank test).

In parallel with affinity and functional profiling at selected molecular targets, preliminary safety screening was undertaken to evaluate the potential cytotoxic effects of the tested compounds. Hepatocytotoxic and neurocytotoxic activity represents one of the most important checkpoints in the development of new potential therapeutic compounds. In order to reduce costs and in line with the 3R principles outline in Directive 2010/63/EU (which promote the replacement of animals with alternatives, reduction in their number, and refinement of procedures to minimize suffering), such analyses are performed at an early stage of development of new compounds using in vitro methods. Here, we performed preliminary studies of hepatocytotoxic and neurocytotoxic activity using two commercially available cell lines—human hepatocellular carcinoma (HepG2) cell line and human neuroblastoma (SHSY-5Y) cell line. We investigated both the effect of the tested compounds on the basic symptom of cytotoxicity, which is cell membrane damage (LDH test), and the metabolic state of the cell, which can also change under the influence of toxic compounds (MTT test). The obtained results indicate that all tested compounds **9**–**22** do not show hepatocytotoxic activity in the lower concentration range (0.5–5 µM) (Figure 4 and Figure S1 in Supplementary Materials). Their toxic activity towards HepG2 cells is manifested in most cases only at a higher concentration of 50 µM. In turn neurocytotoxic activity (Figure 4 and Figure S2 in Supplementary Materials) of **9**–**22** was lower even at highest applied concentration (50 µM) than the reference positive control drug used in the study—doxorubicin (DOX). The results obtained in the LDH assay were reflected also in the MTT viability analysis, as the **9**–**22** IC_50_ values were higher than DOX IC_50_ in both HepG2 and SH-SY5Y cell lines (Table S1 in Supplementary Materials).

The hepatocytotoxic and neurocytotoxic effects of compounds **10** and **20**, which exhibit the highest binding affinity for 5-HT_7_ receptors and potent inhibition of sodium channels, are shown in Figure 4. The viability of HepG2 under the influence of **10** and **20** was promising, with IC_50_ values of 7.55 and 3.15 µM, respectively. In turn, IC_50_ values calculated for **10** and **20** neurotoxic effects were an order of magnitude higher than DOX and amount to 24.63 and 17.23 µM, respectively. Based on the aforementioned results, compound **10** demonstrated a more favorable safety profile than compound **20**, with higher IC_50_ values in both HepG2 cells (7.55 µM) and neuronal cells (24.63 µM).

Considering the results obtained for both compounds **10** and **20** and taking into account the lower cytotoxicity of compound **10** along with its comparable activity toward 5-HT_7_R and sodium channels, this compound was selected for further in vitro and in vivo studies.

### 2.5. In Vitro ADME Studies

The preliminary assessment of compound **10** drug likeness properties was performed with use of following in vitro assays: incubation with human liver microsomes (HLMs) for determination of metabolic stability and the most probable metabolic pathways; determination of drug–drug interactions (DDIs) with use of commercially available luminescent method.

The metabolic stability studies involved in vitro incubation of compound **10** for 120 min with HLMs and the determination of the most probable sites of metabolism with use in silico tool MetaSite 6.0.1 (Figures S3 and S4 in Supplementary Materials). The obtained results are summarized in Table 2.

Compound **10** displayed moderate metabolic stability, with 50.18% of the parent compound remaining in the reaction mixture, compared to only 30.80% for the unstable reference drug Verapamil. LC-MS analysis revealed five main metabolites, with the primary biotransformation pathway identified as monohydroxylation, resulting in metabolite M1. Additional hydroxylated metabolites included M3 (monohydroxylation) and M4 (dihydroxylation). Predicted hydroxylation sites, located in the 1-(2-biphenyl)piperazine moiety and in the tetralin ring, were proposed using MetaSite software (6.0.5) and are shown in Figure S4 (Supplementary Materials). Decomposition of compound **10** was also observed, most likely occurring at the piperazine ring, yielding metabolites M2 and M5 (see Figure S3 in Supplementary Materials). The obtained results indicate slightly improved metabolic stability compared to the rapidly metabolized reference drug Verapamil.

**Figure 5:**
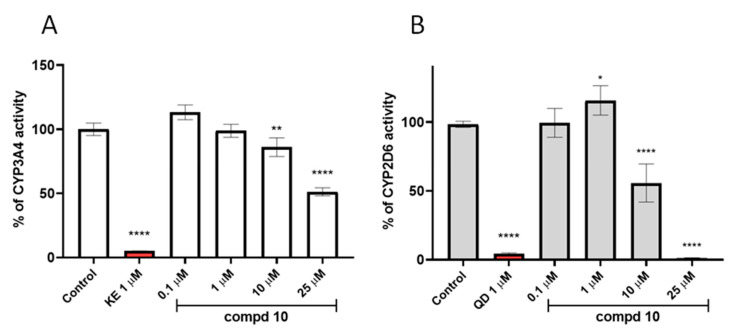
The effect of compound **10** and the reference drugs ketoconazole (KE) and quinidine (QD) on CYP3A4 (**A**), CYP2D6 (**B**) activity. The statistical significance was evaluated using one-way ANOVA and Bonferroni’s multiple comparison post hoc test: * *p* < 0.05, ** *p* < 0.01, **** *p* < 0.0001 (GraphPad Prism 8).

Subsequently, the potential for DDIs involving compound **10** was evaluated. The results revealed that compound **10** exhibits a moderate DDI potential. The most pronounced inhibition was observed for the CYP2D6 isoform, with a statistically significant reduction in enzymatic activity to approximately 50% at 10 µM, and complete inhibition at 25 µM. For CYP3A4, a 50% decrease in activity was observed at 25 µM (see Figure 5).

Considering that compound **10** exhibits high on-target activity (5-HT_7_ binding < 10 nM; 89% sodium channel inhibition at 1 µM) and only moderate CYP inhibition at higher concentrations (≥10 µM), the potential for clinically relevant DDI in humans requires further in vivo investigations.

### 2.6. In Vivo Pharmacology

Selective serotonin 5-HT_7_R ligands have shown antidepressant- and anxiolytic-like effects in preclinical models [34,35]. In addition, sodium channel blockers are used as adjuvants in mood disorders; for example, lamotrigine is clinically applied to enhance antidepressant efficacy in treatment-resistant depression and bipolar disorder [36]. Based on these findings, in the next phase of the study, in vivo experiments were conducted to evaluate the potential antidepressant-like and anxiolytic-like properties of compound **10** in mice. Furthermore, the effect of compound **10** on mechanical and thermal (heat) nociceptive thresholds was also assessed in mice using a STZ-induced diabetic neuropathic pain model, given the involvement of serotonin 5-HT_7_ receptors and voltage-gated sodium channels in pain modulation [37,38]. Moreover, to gain an insight into the role of these two molecular targets in pain sensation, in the STZ model of chronic pain, an ex vivo examination was also conducted on selected tissues whose impaired function is observed in the course of diabetes (i.e., pancreas and sciatic nerve).

#### 2.6.1. Forced Swim Test

**Figure 6:**
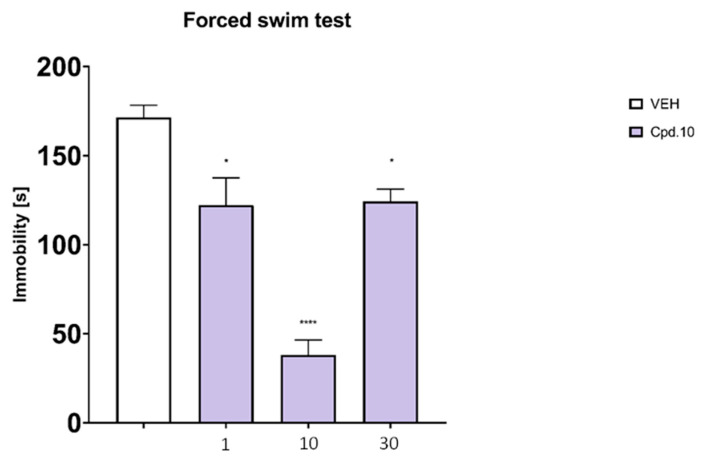
Antidepressant-like activity of the compound **10** used at doses 1, 10 and 30 mg/kg measured using the FST in mice. Results are shown as the mean duration of immobility [s] (±SEM) for n = 8–10. Statistical analysis: one-way ANOVA followed by Dunnett’s post hoc test. Significance vs. control: * *p* < 0.05, **** *p* < 0.0001.

One-way ANOVA and Dunnett’s post hoc test revealed a significant effect of treatment with the compound **10** on immobility duration measured in the FST. Compared to control, compound **10** was effective at all doses tested (1, 10 and 30 mg/kg, *p* < 0.05), in particular at the dose of 10 mg/kg (Figure 6).

#### 2.6.2. Four-Plate Test

**Figure 7:**
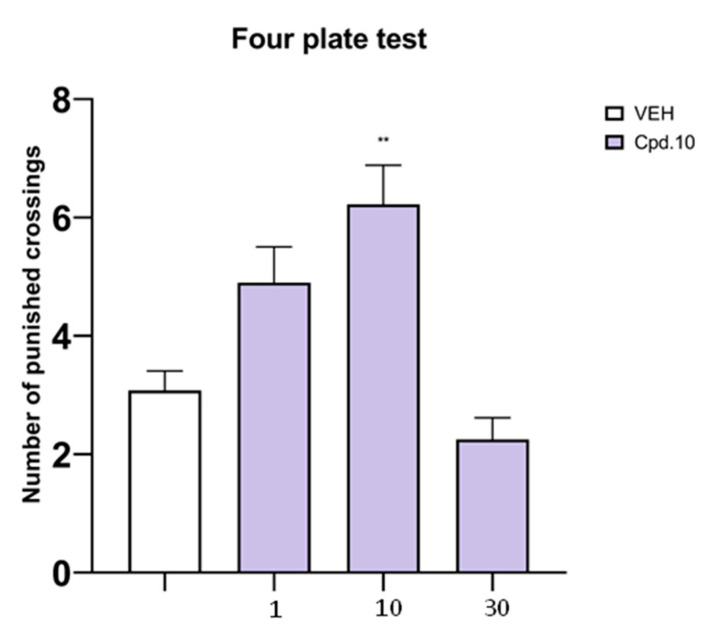
Anxiolytic-like activity of the compound **10** at doses 1, 10 and 30 mg/kg measured using the FPT in mice. Results are shown as the mean number of punished crossings (±SEM) for n = 8–10. Statistical analysis: one-way ANOVA followed by Dunnett’s post hoc test. Significance vs. control: ** *p* < 0.01.

In the mouse FPT a significant effect of treatment with the compound **10** on the number of punished crossings was demonstrated. Dunnett’s post hoc test showed that compared to control **10** increased the number of punished crossings only at the dose of 10 mg/kg (Figure 7).

#### 2.6.3. STZ Model of Painful Peripheral Neuropathy

Similarly to antiepileptic drugs, antidepressants are widely recommended as the first-line treatment of pain in the course of diabetic neuropathy in humans [39]. Although these drugs do not possess direct antidiabetic (i.e., blood glucose-lowering) properties [40,41,42] as they do not reduce blood glucose concentration in patients suffering from neuropathic pain [43], they are used as analgesic adjuvants in this clinical condition, because similar mechanisms are responsible for the antidepressant activity and neuropathic pain relief. Therefore, **10** was also assessed for its potential antiallodynic and antihyperalgesic properties in a mouse model of diabetic neuropathic pain induced by STZ.

In addition to this, a preliminary assessment of the effect of the compound **10** (dose: 10 mg/kg) on blood glucose level, pancreatic architecture and sciatic nerve structure was carried out.

##### Effect on Blood Glucose Level

Considering the above-mentioned issue, the compound **10** was assessed for its effect on blood glucose level in STZ-treated mice. This experiment did not reveal the influence of this compound on this parameter (blood glucose level of vehicle-treated non-diabetic mice: 125.7 ± 0.9 mg/dL; vehicle-treated diabetic mice: 515.7 ± 84.3 mg/dL; diabetic mice treated with the compound **10**: 593.7 ± 6.3).

##### Effect on Mechanical Nociceptive Threshold (Von Frey Test)

**Figure 8:**
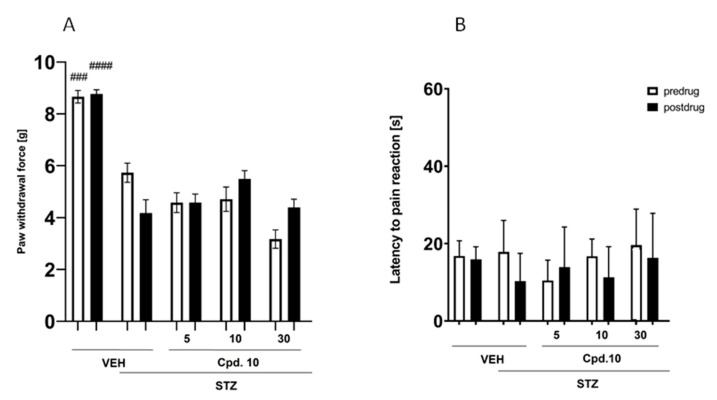
Effect of **10** used at doses 5, 10 and 30 mg/kg on the mechanical (**A**) and heat (**B**) nociceptive threshold assesses in the STZ-induced diabetic neuropathic pain model measured using the von Frey test (**A**) and the hot plate test (**B**), respectively. Results are shown as the mean paw withdrawal threshold [g] (±SEM) in response to mechanical stimulation (**A**), or the mean latency to neuropathic pain reaction [s] (±SEM) in response to thermal stimulation (55–56 °C) (B) for n = 7–10. Statistical analysis: repeated measures analysis of variance (ANOVA), followed by Dunnett’s and Sidak’s post hoc comparisons. VEH bars (pre- and post-drug): left—non-STZ control animals, right—STZ-treated mice. Significance vs. STZ-treated control group (STZ + VEH) at the respective time point of testing: ### *p* < 0.001, #### *p* < 0.0001; significance vs. predrug paw withdrawal threshold, or predrug latency to neuropathic pain reaction measured in the individual group: *p* > 0.05.

In the von Frey test, repeated measures ANOVA revealed an overall significant effect of treatment with the compound **10**. As shown in Figure 8A, the mechanical nociceptive threshold in control mice not treated with STZ was significantly (*p* < 0.001) higher as compared to that of STZ + vehicle-treated control mice. This indicated that in STZ-treated mice mechanical (tactile) allodynia was observed. In diabetic, nauropathic mice, compound **10** at doses 5, 10 and 30 mg/kg did not possess antiallodynic properties in the von Frey test (Figure 8A).

##### Effect on Thermal (Heat) Nociceptive Threshold (Hot Plat Test)

In the mouse model of STZ-induced diabetic neuropathy the hot plate test was used to assess the effect of compound **10** on heat nociceptive threshold. As shown in Figure 8B, there were not significant differences in the latencies to neuropathic pain reaction between the two control groups tested. This indicated that heat hyperalgesia, or heat allodynia did not develop in STZ-exposed mice. In the hot plate test carried out in STZ-treated mice, compound **10** was not effective (Figure 8B).

In summary, the in vivo pharmacological evaluation of compound **10** revealed significant antidepressant-like and anxiolytic-like effects, with the dose of 10 mg/kg showing the most consistent activity across behavioral models. In the FST, compound **10** significantly reduced immobility time at all tested doses, suggesting its antidepressant-like activity. Likewise, in the FPT, a significant increase in the number of punished crossings was observed at 10 mg/kg, indicative of anxiolytic-like effects. These findings are consistent with the compound’s high affinity for the 5-HT_7_ receptor and its proposed CNS mechanism of action.

In contrast, compound **10** did not produce significant antinociceptive effects in the STZ-induced mouse model of diabetic neuropathy, as it failed to reverse mechanical allodynia or affect thermal nociceptive thresholds in the von Frey and hot plate tests, respectively. Despite its activity at sodium channels, these results suggest that compound **10** lacks efficacy in alleviating neuropathic pain in this model. Overall, the findings suggest that compound **10** may hold promise for the treatment of mood disorders, although it did not demonstrate analgesic (and antidiabetic) effects in the neuropathic pain model used in this study.

##### Effect on Pancreatic Cell Architecture and Sciatic Nerve Structure

**Figure 9:**
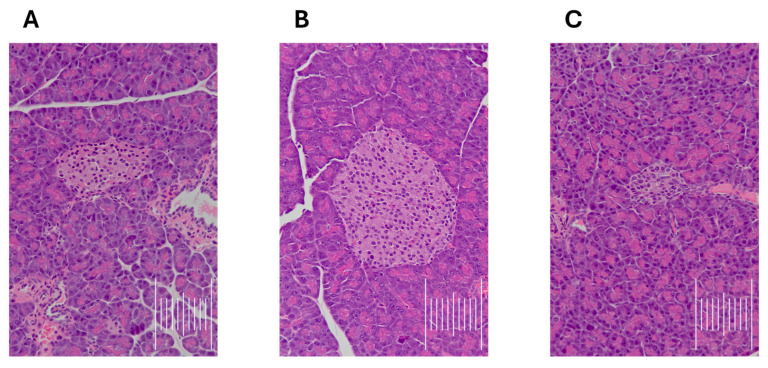
Each photograph shows an example of mouse pancreatic tissue from each group of animals: the pancreatic islet (a group of cells with lighter cytoplasm) is located in the center, surrounded by acini of exocrine glands: (**A**) Control group. (**B**) STZ-Veh group. (**C**) STZ-compd**10** group. Scale in the lower right corners: smallest division—0.01 mm. H&E, obj. magnification: 40×.

In photographs of the pancreases of mice (Figure 9) in the control group, the cross-sectional area of 14 islets was measured, in the STZ-Veh group—16 islets, and in the STZ-compd **10** group—18 islets. The mean cross-sectional area of the islet in the control group was 1.05 mm^2^ (min. 0.17 mm^2^, max. 2.23 mm^2^), in the STZ-Veh group—2.72 mm^2^ (min. 0.61 mm^2^, max. 7.41 mm^2^), and in the STZ-compd**10** group—0.86 mm^2^ (min. 0.28 mm^2^, max. 3.16 mm^2^). The mean size of pancreatic islets in the STZ-compd**10** group was similar to that in the control group, while islets in the STZ-Veh group were on average more than twice as large. Then, pairwise comparisons were performed using the Mann–Whitney U test (for quantitative variables with non-normal distribution). The analysis showed a significant difference between the control group and the STZ-Veh group (*p* = 0.0011), as well as between the STZ-Veh group and the STZ-compd**10** group (*p* = 0.00005). In contrast, no significant difference was observed between the control group and the STZ-compd**10** group (*p* = 0.352). In summary, in the pancreas, islets from STZ-Veh mice were markedly enlarged compared to controls, whereas treatment with compound **10** preserved islet size close to normal.

Slightly increased vacuolization of the cytoplasm of myelin cells and axons was observed in nerve sections in the STZ-Veh and STZ-compd**10** groups compared to the control group of animals. These changes indicate subtle structural alterations in nerves that were absent in controls.

Taken together, the test compound **10** turned out to possess antidepressant-like and anxiolytic-like properties in mice. It has also to be emphasized that in this research preliminary safety pharmacology studies were performed by a thorough observation of animals treated with tested compound **10**. This observation did not reveal any disturbing changes in animals’ behavior (e.g., seizures, motor deficits) after the administration of compound **10**. Moreover, no unexpected adverse events were observed during the experiments, and no animals required early euthanasia. After the completion of behavioral testing, animals were humanely euthanized in compliance with institutional guidelines.

To sum up, this study was designed as a first-in-animal investigation to evaluate the functional activity of newly synthesized compounds in validated behavioral tests/models. At this exploratory stage, our focus was on assessing whether the compound **10** exhibits antidepressant-, anxiolytic-, and/or anti-neuropathic pain-like effects in vivo, rather than elucidating detailed molecular mechanisms.

Limitation of the study. At this stage of exploratory study, first-in-animal work focused on evaluating compound **10**’s in vivo behavioral effects rather than elucidating detailed molecular mechanisms, which may be considered a limitation. Additional limitations include the lack of direct in vivo confirmation of target engagement despite dual in vitro activity, and the absence of mechanistic investigations underlying the observed behavioral effects. Animal experiments were restricted to behavioral testing without complementary biochemical or molecular analyses. Conclusions regarding drug–drug interaction risk were based solely on in vitro data and require further in vivo validation. Nevertheless, as this project is still at the hit-discovery stage of medicinal chemistry, we conducted the studies feasible at this stage of development. Advanced mechanistic studies in mice (e.g., receptor occupancy, signaling pathway analyses, or biomarker profiling) were not included to limit animal use in line with the 3R principle, and because our primary aim was to establish pharmacological proof of concept. Such studies would provide valuable insights, and we plan to address them in future work to strengthen the translational interpretation of these results.

## 3. Materials and Methods

### 3.1. Chemistry

TLC was performed on silica gel 60 F254 aluminum plates (Merck, Darmstadt, Germany), employing two solvent systems: S1—dichloromethane: methanol: ammonia (9:1:2 drops, *v*/*v*); S2—petroleum ether: ethyl acetate (8:2, *v*/*v*); S3—dichloromethane: methanol (9:0.7, *v*/*v*); and S4—dichloromethane: methanol (9:1, *v*/*v*). Visualization of compounds was achieved under UV light at 254 nm. High-performance liquid chromatography (HPLC) analyses were carried out on a Waters system equipped with a photodiode array detector (Waters 2998, Waters, Milford, CT, USA) and a Chromolith SpeedROD RP-18 column (18.5 μm, 4.6 × 50 mm; Merck, Darmstadt, Germany). The mobile phase consisted of eluent A (water with 0.1% TFA) and eluent B (acetonitrile with 0.1% TFA), with a linear gradient from 0 to 100% B over 3 min at a flow rate of 5 mL/min. Standard solutions (1 mg/mL) were prepared in acetonitrile/water (1:1, *v*/*v*) containing 0.1% TFA. Detection was performed over a wavelength range of 200–800 nm. All final compounds exhibited a purity greater than 95%. LC–MS analyses were carried out on a Waters ACQUITY Premier UPLC system coupled with a Xevo TQ-S Cronos mass spectrometer (Waters, Milford, CT, USA) using electrospray ionization (ESI). Chromatographic separation was performed on an Acquity UPLC BEH C18 column (2.1 × 100 mm, 1.7 μm) with a VanGuard pre-column (2.1 × 5 mm, 1.7 μm). Gradient elution was applied using eluent A (water with 0.1% formic acid) and eluent B (acetonitrile with 0.1% formic acid), with a flow rate of 0.3 mL/min and a total run time of 15 min. Samples for HPLC and LC-MS were dissolved in acetonitrile/water (50:50, *v*/*v*) or in pure acetonitrile. Detection was performed using a PDA detector (Waters Corporation, Milford, MA, USA) within a wavelength range of 200–500 nm. NMR spectra were recorded using JEOL (500 MHz, JEOL Ltd., Tokyo, Japan), instruments in CDCl_3_. Tetramethylsilane (TMS) served as the internal standard. Chemical shifts are reported in δ (ppm), with coupling constants (*J*) given in Hz. Signal multiplicities are designated as follows: s (singlet), br. s. (broad singlet), d (doublet), dd (double doublet), t (triplet), dt (doublet of triplets), td (triplet of doublets), and m (multiplet).

All other reagents, including substituted ketones, 1-bromo-3-chloropropane, 1,4-dibromobutane, 1,5-dibromopentane, starting amines, boronic acids, solvents, and inorganic chemicals, were commercially available and obtained from Sigma-Aldrich, Alfa Aesar, or Chemat. Synthetic procedures, as well as physicochemical and spectral data for starting and intermediate imidazolidine-2,4-dione derivatives (**1**–**8**), namely: 3′,4′-dihydro-2′H-spiro[imidazolidine-4,1′-naphthalene]-2,5-dione (**1**), 2′,3′-dihydrospiro[imidazolidine-4,1′-indene]-2,5-dione (**2**), 5-(4-fluorophenyl)-5-methylimidazolidine-2,4-dione (**3**), 1-(5-chloropentyl)-3′,4′-dihydro-2′H-spiro[imidazolidine-4,1′-naphthalene]-2,5-dione (**4**), 1-(4-chlorobutyl)-3′,4′-dihydro-2′H-spiro[imidazolidine-4,1′-naphthalene]-2,5-dione (**5**), 1-(3-chloropropyl)-3′,4′-dihydro-2′H-spiro[imidazolidine-4,1′-naphthalene]-2,5-dione (**6**), 1-(4-chlorobutyl)-2′,3′-dihydrospiro[imidazolidine-4,1′-indene]-2,5-dione (**7**), 3-(4-chlorobutyl)-5-(4-fluorophenyl)-5-methylimidazolidine-2,4-dione (**8**) have been previously described [20,21,22,23]. Three of the 1-(2-biphenyl)piperazine derivatives (**I**, **II**, and **IV**) were previously reported [28,29], while compound **III** was prepared in this study following the same synthetic procedures.

#### 3.1.1. Synthesis of 1-(4′,5-Difluoro-[1,1′-Biphenyl]-2-yl)Piperazine (Compound **III**)

4′,5-Difluoro-[1,1′-biphenyl]-2-amine (3.19 mmol) was dissolved in monomethyl ether of ethylene glycol (1.0 mL), followed by the addition of an equimolar amount of *bis*(2-chloroethyl)amine hydrochloride (3.19 mmol). After being stirred at 150 °C for 24 h, the mixture was cooled, neutralized to pH ~10 with 5% NaOH, and extracted with diethyl ether. The organic extract was dried, concentrated, and purified by column chromatography (solvent system S1).

White solid. Yield: 34%; TLC: R_f_ = 0.38 (S2); HPLC: R_t_ = 1.244 min.; C_16_H_16_F_2_N_2_: calculated *m/z*: 274.31; experimental: [M+H]^+^ = 275.17; ^1^H NMR (500 MHz, CDCl_3_-*d*) δ 1.92–2.08 (m, 1 H) 2.94 (d, *J* = 4.30 Hz, 8 H) 6.91–7.01 (m, 3 H) 7.06–7.12 (m, 2 H) 7.49–7.54 (m, 2 H).

#### 3.1.2. General Procedure for the Synthesis of Final Compounds (**9–22**)

A mixture of the appropriate 3-halogenoalkylhydantoin (0.35 mmol), 1-([1,1′-biphenyl]-2-yl)piperazine derivative (0.42 mmol), potassium carbonate (0.525 mmol), and acetonitrile (2 mL) was placed in a microwave reaction vessel. The reaction was carried out using a CEM Discovery SP Hybrid microwave reactor at 100 °C, 25 W, for 45 min. After completion, the mixture was evaporated under reduced pressure, and the obtained crude was purified through column chromatography (solvent system S3).

- 1-(5-(4-(4′-Fluoro-[1,1′-biphenyl]-2-yl)piperazin-1-yl)pentyl)-3′,4′-dihydro-2′H-spiro[imidazolidine-4,1′-naphthalene]-2,5-dione (compound **9**).

White solid. Yield: 44%; TLC: R_f_ = 0.38 (S3); HPLC: R_t_ = 1.428 min.; C_33_H_37_FN_4_O_2_: calculated *m/z*: 540.68; experimental: [M + H]^+^ = 541.90; ^1^H NMR (500 MHz, CDCl_3_-*d*) δ 1.34 (quin, *J* = 7.52 Hz, 2 H) 1.62–1.86 (m, 5 H) 1.92–2.00 (m, 1 H) 2.18–2.34 (m, 2 H) 2.43–2.92 (m, 8 H) 3.02 (br. s., 4 H) 3.53 (t, *J* = 7.16 Hz, 2 H) 6.08–6.20 (m, 1 H) 6.98–7.23 (m, 9 H) 7.28 (td, *J* = 7.73, 1.72 Hz, 1 H) 7.49–7.58 (m, 2 H); ^13^C NMR (125 MHz, CDCl_3_-*d*) δ 19.15, 24.26, 27.79, 28.87, 34.27, 38.29, 49.40, 52.77, 57.89, 62.78, 115.29 (d, *J* = 21.73 Hz), 118.90, 123.68, 126.45, 127.00, 128.75 (d, *J* = 6.64 Hz), 129.91, 130.51 (d, *J* = 7.84 Hz), 131.44, 133.08, 134.28, 136.76 (d, *J* = 3.02 Hz), 138.25, 156.78, 161.95 (d, *J* = 246.27 Hz), 176.35.

- 1-(4-(4-(4′-Fluoro-[1,1′-biphenyl]-2-yl)piperazin-1-yl)butyl)-3′,4′-dihydro-2′H-spiro[imidazolidine-4,1′-naphthalene]-2,5-dione (compound **10**).

White solid. Yield: 75%; TLC: R_f_ = 0.37 (S3); HPLC: R_t_ = 1.410 min.; C_32_H_35_FN_4_O_2_: calculated *m/z*: 526.66; experimental: [M + H]^+^ = 527.31; ^1^H NMR (500 MHz, CDCl_3_-*d*) δ 1.50 (quin, *J* = 7.52 Hz, 2 H) 1.66 (quin, *J* = 7.37 Hz, 2 H) 1.74–1.83 (m, 1 H) 1.91–1.98 (m, 1 H) 2.19–2.44 (m, 8 H) 2.77–2.91 (m, 6 H) 3.54 (t, *J* = 7.16 Hz, 2 H) 6.13 (br. s., 1 H) 6.99–7.03 (m, 2 H) 7.03–7.14 (m, 5 H) 7.17–7.22 (m, 2 H) 7.25–7.30 (m, 1 H) 7.56–7.61 (m, 2 H); ^13^C NMR (125 MHz, CDCl_3_-*d*) δ 19.15, 23.84, 26.19, 28.88, 34.26, 38.59, 50.86, 53.30, 58.01, 62.73, 115.07 (d, *J* = 20.52 Hz), 118.53, 122.89, 126.49, 126.97, 128.53, 128.78, 129.91, 130.54 (d, *J* = 7.84 Hz), 131.40, 133.09, 134.08, 137.09 (d, *J* = 3.62 Hz), 138.22, 150.24, 156.88, 161.89 (d, *J* = 243.86 Hz), 176.28.

- 1-(3-(4-(4′-Fluoro-[1,1′-biphenyl]-2-yl)piperazin-1-yl)propyl)-3′,4′-dihydro-2′H-spiro[imidazolidine-4,1′-naphthalene]-2,5-dione (compound **11**).

White solid. Yield: 55%; TLC: R_f_ = 0.34 (S3); HPLC: R_t_ = 1.403 min.; C_31_H_33_FN_4_O_2_: calculated *m/z*: 512.26; experimental: [M + H]^+^ = 513.35; ^1^H NMR (500 MHz, CDCl_3_-*d*) δ 1.75–1.83 (m, 1 H) 1.89 (br. s., 2 H) 1.94–2.00 (m, 1 H) 2.16–2.35 (m, 3 H) 2.45 (br. s., 5 H) 2.77–2.91 (m, 6 H) 3.59 (t, *J* = 7.16 Hz, 2 H) 6.21 (br. s., 1 H) 6.98–7.03 (m, 2 H) 7.04–7.09 (m, 3 H) 7.11–7.16 (m, 2 H) 7.17–7.23 (m, 2 H) 7.25–7.30 (m, 1 H) 7.54–7.59 (m, 2 H); ^13^C NMR (125 MHz, CDCl_3_-*d*) δ 19.16, 25.24, 28.87, 34.17, 36.88, 50.44, 53.19, 55.56, 62.76, 115.18 (d, *J* = 21.20 Hz), 118.62, 126.47, 126.97, 128.57, 128.79, 129.91, 130.52 (d, *J* = 7.80 Hz), 131.40, 133.08, 134.17, 136.99 (d, *J* = 3.30 Hz), 138.22, 156.71, 161.91 (d, *J* = 245.70 Hz) 176.27.

- 1-(4-(4-([1,1′-Biphenyl]-2-yl)piperazin-1-yl)butyl)-3′,4′-dihydro-2′H-spiro[imidazolidine-4,1′-naphthalene]-2,5-dione (compound **12**).

White solid. Yield: 75%; TLC: R_f_ = 0.33 (S3); HPLC: R_t_ = 1.395 min.; C_32_H_36_N_4_O_2_: calculated *m/z*: 508.67; experimental: [M + 2H]^+^ = 510.90; ^1^H NMR (500 MHz, CDCl_3_-*d*) δ 1.45–1.56 (m, 2 H) 1.65 (quin, *J* = 7.45 Hz, 2 H) 1.73–1.83 (m, 1 H) 1.95 (ddd, *J* = 13.25, 8.66, 3.01 Hz, 1 H) 2.19–2.46 (m, 8 H) 2.77–2.92 (m, 6 H) 3.48–3.59 (m, 2 H) 6.17 (br. s., 1 H) 6.97–7.15 (m, 5 H) 7.17–7.31 (m, 4 H) 7.35–7.42 (m, 2 H) 7.61 (dd, *J* = 8.16, 1.29 Hz, 2 H); ^13^C NMR (125 MHz, CDCl_3_-*d*) δ 19.14, 23.75, 26.18, 28.88, 34.26, 38.56, 50.71, 53.25, 58.00, 62.73, 118.32, 122.75, 126.49, 126.84, 126.98, 128.28, 128.40, 128.77, 128.93, 129.89, 131.55, 133.10, 134.99, 138.22, 141.23, 150.16, 156.88, 176.29.

- 1-(4-(4-(4′,5-Difluorobiphen-2-yl)piperazin-1-yl)butyl)-3′,4′-dihydro-2′H-spiro[imidazolidine-4,1′-naphthalene]-2,5-dione (compound **13**).

White solid. Yield: 79%; TLC: R_f_ = 0.45 (S3); HPLC: R_t_ = 1.541 min.; C_32_H_34_F_2_N_4_O_2_: calculated *m/z*: 544.65; experimental: [M + H]^+^ = 545.38; ^1^H NMR (500 MHz, CDCl_3_-*d*) δ 1.48 (quin, *J* = 7.59 Hz, 2 H) 1.65 (quin, *J* = 7.45 Hz, 2 H) 1.73–1.84 (m, 1 H) 1.90–1.99 (m, 1 H) 2.18–2.42 (m, 8 H) 2.69–2.93 (m, 6 H) 3.54 (t, *J* = 7.16 Hz, 2 H) 6.02–6.15 (m, 1 H) 6.89–6.97 (m, 3 H) 7.00 (d, *J* = 7.45 Hz, 1 H) 7.04–7.15 (m, 4 H) 7.16–7.23 (m, 1 H) 7.53–7.61 (m, 2 H); ^13^C NMR (125 MHz, CDCl_3_-*d*) δ 19.14, 23.95, 26.20, 28.87, 34.27, 38.63, 51.34, 53.32, 58.01, 62.72, 114.41 (d, *J* = 21.13 Hz), 115.13 (d, *J* = 21.13 Hz), 117.85 (d, *J* = 22.94 Hz), 119.91 (d, *J* = 8.45 Hz), 126.48, 126.94, 128.76, 129.89, 130.59 (d, *J* = 7.85 Hz), 133.12, 135.81 (d, *J* = 3.60 Hz), 135.83 (d, *J* = 3.02 Hz), 136.04 (d, *J* = 7.24 Hz), 138.22, 146.51, 156.88, 158.87 (d, *J* = 242.04 Hz), 162.14 (d, *J* = 246.27 Hz), 176.25.

- 1-(4-(4-(5-Fluoro-4′-methoxybiphen-2-yl)piperazin-1-yl)butyl)-3′,4′-dihydro-2′H-spiro[imidazolidine-4,1′-naphthalene]-2,5-dione (compound **14**).

White solid. Yield: 63%; TLC: R_f_ = 0.44 (S3); HPLC: R_t_ = 1.523 min.; C_33_H_37_FN_4_O_3_: calculated *m/z*: 556,68; experimental: [M + H]^+^ = 557.54; ^1^H NMR (500 MHz, CDCl_3_-*d*) δ 1.42–1.56 (m, 2 H) 1.60–1.70 (m, 2 H) 1.73–1.84 (m, 1 H) 1.89–1.99 (m, 1 H) 2.18–2.41 (m, 8 H) 2.71–2.92 (m, 6 H) 3.55 (t, *J* = 7.02 Hz, 2 H) 3.84 (s, 3 H) 5.87 (br. s., 1 H) 6.84–6.97 (m, 5 H) 7.01 (d, *J* = 7.73 Hz, 1 H) 7.09–7.15 (m, 2 H) 7.18–7.23 (m, 1 H) 7.52–7.58 (m, 2 H); ^13^C NMR (125 MHz, CDCl_3_-*d*) δ 19.16, 23.99, 26.22, 28.87, 34.30, 38.68, 51.26, 53.40, 55.33, 58.07, 62.71, 113.68, 113.78 (d, *J* = 21.13 Hz) 117.78 (d, *J* = 22.33 Hz), 119.58 (d, *J* = 7.85 Hz), 126.48, 126.98, 128.79, 129.90, 130.00, 132.34, 133.08, 136.59, 136.65, 138.22, 146.48, 158.34 (d, *J* = 240.23 Hz), 176.20.

- 1-(4-(4-(4′-Fluoro-[1,1′-biphenyl]-2-yl)piperazin-1-yl)butyl)-2′,3′-dihydrospiro[imidazolidine-4,1′-indene]-2,5-dione (compound **15**).

Creamy solid. Yield: 58%; TLC: R_f_ = 0.42 (S3); HPLC: R_t_ = 1.458 min.; C_31_H_33_FN_4_O_2_: calculated *m/z*: 526.66; experimental: [M+H]^+^ = 513.50; ^1^H NMR (500 MHz, CDCl_3_-*d*) 1.44–1.54 (m, 2 H) 1.66 (dt, *J* = 14.89, 7.45 Hz, 3 H) 2.18–2.44 (m, 7 H) 2.70 (ddd, *J* = 13.25, 8.38, 3.58 Hz, 1 H) 2.83 (br. s., 4 H) 3.04 (ddd, *J* = 15.97, 8.95, 3.44 Hz, 1 H) 3.19–3.31 (m, 1 H) 3.54 (t, *J* = 7.16 Hz, 2 H) 5.60 (br. s., 1 H) 6.99–7.11 (m, 5 H) 7.15–7.22 (m, 2 H) 7.27–7.34 (m, 2 H) 7.54–7.62 (m, 2 H); ^13^C NMR (125 MHz, CDCl_3_-*d*) δ 23.88, 26.16, 30.26, 37.10, 38.56, 51.31, 53.30, 57.97, 71.30, 114.42 (d, *J* = 21.80 Hz), 115.21 (d, *J* = 21.00 Hz), 117.85 (d, *J* = 22.40 Hz), 119.92 (d, *J* = 8.45 Hz), 122.64, 125.61, 127.44, 129.72, 130.58 (d, *J* = 7.85 Hz), 136.04 (d, *J* = 7.84 Hz), 140.06, 156.93, 158.88 (d, *J* = 245.00 Hz), 161.15, 163.11, 175.57.

- 1-(4-(4-([1,1′-Biphenyl]-2-yl)piperazin-1-yl)butyl)-2′,3′-dihydrospiro[imidazolidine-4,1′-indene]-2,5-dione (compound **16**).

Creamy solid. Yield: 68%; TLC: R_f_ = 0.31 (S4); HPLC: R_t_ = 1.433 min.; C_31_H_34_N_4_O_2_: calculated *m/z*: 494.64; experimental: [M+H]^+^ = 495.54; ^1^H NMR (500 MHz, CDCl_3_-*d*) δ 1.43–1.52 (m, 2 H) 1.64 (quin, *J* = 7.37 Hz, 2 H) 2.14–2.40 (m, 7 H) 2.68 (dddd, *J* = 13.39, 8.38, 3.44, 1.43 Hz, 1 H) 2.83 (br. s., 4 H) 3.03 (ddd, *J* = 15.90, 9.02, 3.44 Hz, 1 H) 3.23 (dt, *J* = 16.11, 8.13 Hz, 1 H) 3.52 (t, *J* = 7.02 Hz, 2 H) 5.81 (br. s., 1 H) 6.99–7.12 (m, 3 H) 7.16–7.24 (m, 2 H) 7.25–7.31 (m, 4 H) 7.34–7.41 (m, 2 H) 7.58–7.64 (m, 2 H); ^13^C NMR (125 MHz, CDCl_3_-*d*) δ 23.89, 26.18, 30.27, 37.13, 38.58, 50.86, 53.30, 58.05, 71.30, 118.28, 122.66, 125.60, 126.81, 127.47, 128.26, 128.38, 128.93, 129.72, 131.54, 134.97, 140.05, 141.26, 144.20, 150.26, 156.89, 175.55.

- 1-(4-(4-(4′,5-Difluorobiphen-2-yl)piperazin-1-yl)butyl)-2′,3′-dihydrospiro[imidazolidine-4,1′-indene]-2,5-dione (compound **17**).

Creamy solid. Yield: 62%; TLC: R_f_ = 0.57 (S4); HPLC: R_t_ = 1.489 min.; C_31_H_32_F_2_N_4_O_2_: calculated *m/z*: 530.62; experimental: [M + H]^+^ = 531.29; ^1^H NMR (500 MHz, CDCl_3_-*d*) δ 1.41–1.51 (m, 2 H) 1.64 (quin, *J* = 7.37 Hz, 2 H) 2.16–2.41 (m, 7 H) 2.63–2.71 (m, 1 H) 2.75 (t, *J* = 4.58 Hz, 4 H) 3.03 (ddd, *J* = 16.04, 8.88, 3.44 Hz, 1 H) 3.23 (dt, *J* = 15.97, 8.20 Hz, 1 H) 3.52 (t, *J* = 7.02 Hz, 2 H) 5.87–6.03 (m, 1 H) 6.90–6.99 (m, 3 H) 7.04–7.11 (m, 3 H) 7.17–7.22 (m, 1 H) 7.27–7.32 (m, 2 H) 7.54–7.60 (m, 2 H); ^13^C NMR (125 MHz, CDCl_3_-*d*) δ 23.88, 26.16, 30.26, 37.13, 38.56, 51.31, 53.30, 57.97, 71.30, 114.42 (d, *J* = 21.60 Hz), 115.21 (d, *J* = 21.60 Hz), 117.85 (d, *J* = 22.60 Hz), 119.92 (d, *J* = 8.45 Hz),122.64, 125.61, 127.44, 129.72, 130.58 (d, *J* = 7.85 Hz), 136.04 (d, *J* = 7.84 Hz), 140.06, 144.21, 146.48 (d, *J* = 2.42 Hz), 156.93, 158.88 (d, *J* = 241.50 Hz),162.13 (d, *J* = 246.90 Hz), 175.57.

- 1-(4-(4-(5-Fluoro-4′-methoxybiphen-2-yl)piperazin-1-yl)butyl)-2′,3′-dihydrospiro[imidazolidine-4,1′-indene]-2,5-dione (compound **18**).

Creamy solid. Yield: 59%; TLC: R_f_ = 0.53 (S3); HPLC: R_t_ = 1.422 min.; C_31_H_35_FN_4_O_3_: calculated *m/z*: 542.66; experimental: [M+H]^+^ = 543.39; ^1^H NMR (500 MHz, CDCl_3_-*d*) δ 1.43–1.53 (m, 2 H) 1.59–1.70 (m, 2 H) 2.17–2.44 (m, 7 H) 2.68 (d, *J* = 3.72 Hz, 1 H) 2.77 (br. s., 3 H) 2.98–3.07 (m, 1 H) 3.18–3.29 (m, 1 H) 3.53 (t, *J* = 7.16 Hz, 2 H) 3.80–3.87 (m, 3 H) 5.80–5.91 (m, 1 H) 6.88–6.98 (m, 6 H) 7.08 (d, *J* = 7.45 Hz, 1 H) 7.17–7.21 (m, 1 H) 7.28–7.33 (m, 2 H) 7.52–7.57 (m, 2 H); ^13^C NMR (125 MHz, CDCl_3_-*d*) δ 23.86, 26.17, 30.27, 37.15, 38.57, 51.18, 53.37, 55.33, 58.01, 71.30, 113.68, 113.80 (d, *J* = 21.00 Hz), 117.78 (d, *J* = 22.50 Hz), 119.60 (d, *J* = 7.85 Hz), 122.65, 125.61, 127.46, 129.72, 130.00, 132.33, 136.62 (d, *J* = 7.24 Hz), 140.04, 144.21, 146.44, 156.87, 158.87 (d, *J* = 243.00 Hz), 158.85, 175.55.

- 3-(4-(4-(4′-Fluoro-[1,1′-biphenyl]-2-yl)piperazin-1-yl)butyl)-5-(4-fluorophenyl)-5-methylimidazolidine-2,4-dione (compound **19**).

White solid. Yield: 61%; TLC: R_f_ = 0.41 (S3); HPLC: R_t_ = 1.492 min.; C_30_H_32_F_2_N_4_O_2_: calculated *m/z*: 518.61; experimental: [M + H]^+^ = 519.39; ^1^H NMR (500 MHz, CDCl_3_-*d*) δ 1.41 (quin, *J* = 7.66 Hz, 2 H) 1.56–1.64 (m, 2 H) 1.79 (s, 3 H) 2.17–2.41 (m, 6 H) 2.75–2.84 (m, 4 H) 3.49 (td, *J* = 7.16, 2.00 Hz, 2 H) 6.11–6.26 (m, 1 H) 6.98–7.11 (m, 6 H) 7.19 (dd, *J* = 7.45, 1.72 Hz, 1 H) 7.26–7.30 (m, 1 H) 7.42–7.49 (m, 2 H) 7.55–7.61 (m, 2 H); ^13^C NMR (125 MHz, CDCl_3_-*d*) δ 23.83, 25.83, 26.05, 38.76, 50.92, 53.30, 57.96, 63.13, 115.11 (d, *J* = 21.13 Hz), 115.18, 115.80 (d, *J* = 21.13 Hz), 118.48, 122.84, 127.21 (d, *J* = 8.45 Hz), 128.52, 130.53 (d, *J* = 7.84 Hz), 131.39, 134.09, 134.60 (d, *J* = 3.62 Hz), 137.09 (d, *J* = 3.62 Hz), 150.28, 161.88 (d, *J* = 245.06 Hz), 162.79 (d, *J* = 247.48 Hz), 175.15.

- 3-(4-(4-([1,1′-Biphenyl]-2-yl)piperazin-1-yl)butyl)-5-(4-fluorophenyl)-5-methylimidazolidine-2,4-dione (compound **20**).

White solid. Yield: 49%; TLC: R_f_ = 0.38 (S4); HPLC: R_t_ = 1.464 min.; C_30_H_33_FN_4_O_2_: calculated *m/z*: 500.62; experimental: [M + H]^+^ = 501.38; ^1^H NMR (500 MHz, CDCl_3_-*d*) δ 1.38–1.47 (m, 2 H) 1.59 (quin, *J* = 7.52 Hz, 2 H) 1.78 (s, 3 H) 2.19–2.45 (m, 6 H) 2.84 (t, *J* = 4.30 Hz, 4 H) 3.48 (td, *J* = 7.23, 2.15 Hz, 2 H) 6.48 (br. s., 1 H) 6.97–7.10 (m, 4 H) 7.20–7.23 (m, 1 H) 7.26–7.30 (m, 2 H) 7.34–7.40 (m, 2 H) 7.42–7.49 (m, 2 H) 7.56–7.63 (m, 2 H); ^13^C NMR (125 MHz, CDCl_3_-*d*) δ 23.56, 25.87, 26.01, 38.66, 50.63, 53.15, 57.83, 63.17, 115.88 (d, *J* = 21.73 Hz), 118.29, 122.78, 126.83, 127.22 (d, *J* = 8.45 Hz), 128.28, 128.41, 128.93, 131.55, 134.61 (d, *J* = 3.62 Hz), 134.99, 141.22, 150.10, 156.78, 162.78 (d, *J* = 247.48 Hz), 175.20.

- 3-(4-(4-(4′,5-Difluorobiphen-2-yl)piperazin-1-yl)butyl)-5-(4-fluorophenyl)-5-methylimidazolidine-2,4-dione (compound **21**).

White solid. Yield: 75%; TLC: R_f_ = 0.45 (S4); HPLC: R_t_ = 1.509 min.; C_30_H_31_F_3_N_4_O_2_: calculated *m/z*: 536.60; experimental: [M + H]^+^ = 537.27; ^1^H NMR (500 MHz, CDCl_3_-*d*) δ 1.35–1.45 (m, 2 H) 1.59 (quin, *J* = 7.52 Hz, 2 H) 1.78 (s, 3 H) 2.18–2.44 (m, 6 H) 2.73 (t, *J* = 4.01 Hz, 4 H) 3.46–3.51 (m, 2 H) 6.43–6.52 (m, 1 H) 6.88–6.98 (m, 3 H) 7.00–7.12 (m, 4 H) 7.42–7.49 (m, 2 H) 7.52–7.60 (m, 2 H); ^13^C NMR (125 MHz, CDCl_3_-*d*) δ 23.85, 25.88, 26.09, 38.74, 51.32, 53.27, 57.90, 63.15, 114.42 (d, *J* = 21.73 Hz), 115.20 (d, *J* = 21.13 Hz),115.85 (d, *J* = 21.73 Hz), 117.76, 117.94, 119.88 (d, *J* = 8.45 Hz), 127.20 (d, *J* = 7.85 Hz,) 130.58 (d, *J* = 7.85 Hz), 134.63 (d, *J* = 3.62 Hz), 136.02, 136.08, 146.46, 156.80, 158.88 (d, *J* = 242.65 Hz), 162.44 (dd, *J* = 248.68, 247.48 Hz), 175.18.

- 3-(4-(4-(5-Fluoro-4′-methoxybiphen-2-yl)piperazin-1-yl)butyl)- 5-(4-fluorophenyl)-5-methylimidazolidine-2,4-dione (compound **22**).

White solid. Yield: 54%; TLC: R_f_ = 0.49 (S1); HPLC: R_t_ = 1.495 min.; C_31_H_34_F_2_N_4_O_3_: calculated *m/z*: 548.63; experimental: [M + H]^+^ = 549.32; ^1^H NMR (500 MHz, CDCl_3_-*d*) δ 1.37–1.46 (m, 2 H) 1.59 (quin, *J* = 7.45 Hz, 2 H) 1.78 (s, 3 H) 2.18–2.42 (m, 6 H) 2.75 (br. s., 4 H) 3.48 (td, *J* = 6.95, 2.43 Hz, 2 H) 3.84 (s, 3 H) 6.25–6.38 (m, 1 H) 6.86–6.97 (m, 5 H) 7.01–7.07 (m, 2 H) 7.41–7.49 (m, 2 H) 7.51–7.57 (m, 2 H); ^13^C NMR (125 MHz, CDCl_3_-*d*) δ 23.71, 25.84, 26.02, 38.72, 51.13, 53.30, 55.33, 63.14, 113.68, 113.81 (d, *J* = 21.73 Hz), 115.87 (d, *J* = 21.73 Hz), 117.79, 119.58, 127.21 (d, *J* = 8.45 Hz), 130.01, 132.33, 134.61 (d, *J* = 3.62 Hz), 136.63, 146.41, 156.73, 158.87, 158.83, 162.78 (d, *J* = 246.27 Hz), 175.17.

### 3.2. Functional and Radioligand Binding Assay

#### 3.2.1. Radioligand Binding Assay

Affinity of the compounds for the 5-HT_7_ receptor was determined in radioligand binding assays using [^3^H]5-CT as the reference ligand. HEK293 cells stably expressing human 5-HT_7_R (generated with Lipofectamine 2000, Invitrogen, Carlsbad, CA, USA) were cultured under standard conditions (37 °C, 5% CO_2_, DMEM supplemented with 10% dialyzed FBS and 500 µg/mL G418). Cell membranes were prepared from confluent cultures by washing, pelleting, freezing, and subsequent homogenization in assay buffer, followed by repeated centrifugation steps. The assay buffer contained 50 mM Tris-HCl, 4 mM MgCl_2_, 10 mM pargyline, and 0.1% ascorbate. Binding reactions (200 µL) were carried out in 96-well plates for 1 h at 37 °C and terminated by rapid filtration. Radioactivity retained on the filters was measured with a Microbeta TopCount counter (PerkinElmer, Waltham, MA, USA). Non-specific binding was defined with 10 µM 5-HT. Inhibition constants (K_i_) were calculated according to the Cheng–Prusoff equation [44].

Voltage-gated sodium channel binding was assessed under contract at Eurofins-CEREP (Poitiers, France) using their validated site-2 radioligand competition protocol; further methodological details have been described elsewhere [45].

#### 3.2.2. Functional Assay for 5-HT_7_R

The functional properties of compounds in HEK293 cells overexpressing 5-HT_7_R were evaluated for their ability to increase cAMP production for the agonists or to inhibit 10 nM 5-CT at a concentration producing 80% (EC_80_) of the maximum agonist activation for the antagonists. HEK293 cells stably expressing the receptor (generated with Lipofectamine 2000) were cultured at 37 °C in a humidified 5% CO_2_ atmosphere in DMEM supplemented with 10% dialyzed FBS and 500 µg/mL G418. For functional studies, cells were expanded in 25 cm^2^ flasks to ~90% confluence, washed with prewarmed PBS, and centrifuged (5 min, 160× *g*). The pellets were resuspended in stimulation buffer (HBSS with 5 mM HEPES, 0.5 mM IBMX, and 0.1% BSA). Intracellular cAMP levels were quantified using the LANCE^®^ Ultra cAMP detection kit (PerkinElmer, Waltham, MA, USA) according to the manufacturer’s protocol. Briefly, cells (5 µL) were incubated with test compounds (5 µL) for 30 min at room temperature in 384-well plates, followed by addition of detection reagents (Eu-cAMP and ULight-anti-cAMP, 5 µL each). After 1 h, TR-FRET signals were recorded using an Infinite M1000 Pro reader (Tecan, Männedorf, Switzerland).

### 3.3. Molecular Modeling Studies

The model of the 5-HT_7_R was built using the DMFold platform [46]. The following tasks were performed using the Small-Molecule Drug Discovery Suite 2024-4 (Schrödinger Inc., New York, NY, USA), on a workstation running the Linux Ubuntu 24.04 LTS operating system. The receptor structure was refined by docking risperidone into the orthosteric binding site (constrains on Asp3.32) using Induced-Fit Docking (IFD) protocol. To ensure the accuracy of the docking, the resulting complexes were compared with known experimental structures of the 5-HT_7_ receptor (PDB: 7XTC) and a complex of risperidone with 5HT_2A_ receptor (PDB: 6A93), in order to verify ligand positioning and interactions. One of the most active novel ligands, **10**, was docked using the standard IFD protocol to allow the binding site to adapt to larger ligands, and the best-scoring complex served as the source of protein model for subsequent docking. The final poses resulted from Glide SP docking, with the grid box center and constraints set on Asp3.32.

### 3.4. Cytotoxicity Analysis

Cytotoxicity analysis was performed using two established human cell lines: neuroblastoma SH-SY5Y (as a model of neurocytotoxicity; ATCC^®^, CRL-2266™) and hepatocellular carcinoma HepG2 (as a model of hepatocytotoxicity; ATCC^®^ HB-8065™). Cells were cultured under standard conditions in Dulbecco’s Modified Eagle Medium (DMEM) supplemented with 10% fetal bovine serum (FBS) and 1% penicillin–streptomycin. Cultures were maintained at 37 °C in a humidified atmosphere containing 5% CO_2_. For cytotoxicity assays, cells were seeded into 96-well plates at a density of 1 × 10^4^ cells/well and allowed to adhere overnight. After 24 h, the culture medium was replaced with fresh medium containing the test compounds at concentrations ranging from 0.5 to 50 µM. Cells were subsequently incubated for an additional 24 h. To assess cytotoxic effects related to plasma membrane integrity, the CyQUANT™ LDH Cytotoxicity Assay Kit (Thermo Fisher Scientific, Waltham, MA, USA) was used. LDH release into the culture medium, indicative of cell membrane damage, was quantified after 24 h of exposure to the test compounds. Maximum LDH activity (100% cytotoxicity) was determined using the kit’s positive control according to the manufacturer’s instructions. Cell viability was assessed using the MTT (3-(4,5-dimethylthiazol-2-yl)-2,5diphenyltetrazolium bromide) (Sigma-Aldrich, St. Louis, MO, USA) assay, which measures the metabolic activity of viable cells via reduction in MTT to formazan; absorbance was recorded at 570 nm using a microplate reader (SpectraMax iD3, Molecular Devices, San Jose, CA, USA). A higher optical density correlates with increased cell viability. IC_50_ values were calculated based on MTT assay results using nonlinear regression analysis in GraphPad Prism 10 software (CA, USA). All experiments were conducted in triplicate across three independent biological replicates. Results are presented as mean ± SEM and expressed as percent viability (MTT assay) or percent cytotoxicity (LDH assay), corresponding to neurocytotoxicity in SH-SY5Y cells and hepatocytotoxicity in HepG2 cells.

### 3.5. In Vitro ADMET Studies

Metabolic stability studies were performed in silico and in vitro. The in silico metabolism prediction was performed by MetaSite 6.0.1 software (Molecular Discovery Ltd., Hertfordshire, UK). In vitro assays involved the use of human liver microsomes (HLMs) delivered by Sigma-Aldrich (St. Louis, MO, USA). In brief, compound **10** (20 µM) was incubated with HLMs (2 mg/mL) for 120 min in TRIS buffer (pH 7.4) in the presence of NADPH Regeneration System (Promega, Madison, WI, USA). The reaction mixture was incubated for 120 min, terminated next by cold methanol and centrifuged. The supernatant was analyzing by UPLC for estimating % remaining of the substrate. The metabolites were identified also by MS. Verapamil, metabolically unstable control, was tested and analyzed under the same conditions.

The influence on activity of recombinant human cytochromes CYP3A4 and CYP2D6 were measured luminescently with the use of respective P450-Glo™ kits provided by Promega (Madison, WI, USA). The tests were performed according to the protocol provided by the manufacturer. Compound **10** was tested in the concentration range 0.1–25 µM. The ketoconazole and quinidine (the working concentration 1 µM) were used as reference inhibitors and purchased from Sigma-Aldrich (St. Louis, MO, USA). Luminescence signal was measured with a microplate reader (Tecan Spark^®^, Tecan Group Ltd., Maennedorf, Switzerland). Statistical significances were calculated by GraphPad Prism 8.0 software.

### 3.6. Pharmacology In Vivo

#### 3.6.1. Animals and Housing Conditions

Adult male Albino Swiss (CD-1) mice (18–22 g) were obtained from the Animal Breeding Facility of the Faculty of Pharmacy, Jagiellonian University Medical College (Krakow, Poland). Animals were kept in cages (10 mice per cage) under controlled conditions (22 ± 2 °C, 55 ± 10% humidity, 12 h light/dark cycle, lights on at 7:00). Food and water were provided ad libitum. Specified conditions for the maintenance of mice were ensured throughout the experiments, including tree bedding (Transwior, Kotlin, Poland) and cage enrichment (tunnels, nesting material, wooden igloos, etc.). For the behavioral test, the mice were selected randomly. In the experiments, each group consisted of 7–10 mice. The behavioral assays were performed between 9 AM and 3 PM. Following the in vivo experiments, mice were euthanized. All procedures were approved by the 1st Local Ethics Committee of the Jagiellonian University, Krakow (Approval No. 336/2019), and conducted in accordance with Polish regulations and EU Directive 2010/63/EU.

#### 3.6.2. Drugs and Dose Selection for the In Vivo Tests

The tested compound **10** was synthesized at the Chair of Pharmaceutical Chemistry, Faculty of Pharmacy, Jagiellonian University Medical College. Before in vivo assays the compound was suspended in 1% Tween 80 (Sigma Aldrich, Poznań, Poland) and was administered to mice by the intraperitoneal route (i.p.) 60 min before the forced swim test (FST), four-plate test (FPT), and 60 min before the postdrug measurements in neuropathic pain tests (von Frey and hot plate tests). Doses of test compound were selected experimentally, i.e., the dose of 30 mg/kg was a starting dose. This dose was chosen as an initial one because our previous studies of other compounds and reference drugs (e.g., pregabalin) also focused on this dose (for details please see example in [47]). Therefore, in order to reliably assess the effect of the test compound **10** and to compare its effectiveness to available drugs in behavioral tests, this dose was adopted as the starting dose. If it was active, lower doses were subsequently evaluated for their biological activity. Control animals were given an appropriate amount of vehicle (1% Tween 80). STZ used for the induction of painful diabetic neuropathy was supplied by Sigma Aldrich (Poznań, Poland).

#### 3.6.3. Behavioral Tests

##### Forced Swim Test (Assessment of Antidepressant-Like Activity)

The forced swim test was performed according to the procedure of Porsolt et al. [48] with minor adjustments. Mice were placed individually in glass cylinders (25 cm height, 10 cm diameter) filled with 11 cm of water maintained at 23–25 °C for a total of 6 min. Immobility time was recorded during the last 4 min of the session. Animals were considered immobile when floating passively, making only minimal movements to keep the head above water. Reduced duration of immobility was a measure of a compound’s antidepressant-like properties.

##### Four-Plate Test (Assessment of Anxiolytic-Like Activity)

The four-plate apparatus (Bioseb, France) consists of a cage (25 × 18 × 16 cm) with a floor composed of four rectangular metal plates (11 × 8 cm) separated by 4 mm gaps and connected to an electroshock generator. The procedure followed the method of Bourin et al. (2005) [49]. After a 15 s habituation period, mice received a mild electric shock (0.8 mA, 0.5 s) upon crossing between plates (two paws on each plate). Punished crossings were counted for 60 s, and an increased number of such crossings was considered indicative of anxiolytic-like activity.

##### STZ-Induced Painful Diabetic Neuropathy Model (Assessment of Antiallodynic and Antihyperalgesic Activities)

Induction of Neuropathy—STZ Model (Diabetic Neuropathy Model)

Type I diabetes was induced in mice by intraperitoneal administration of streptozotocin (STZ, 200 mg/kg; Polskie Odczynniki Chemiczne, Gliwice, Poland) dissolved in 0.1 N citrate buffer. Age-matched controls received the same volume of citrate buffer. Twenty days after STZ treatment, blood glucose concentration was determined using a blood glucose monitoring system (AccuChek Active, Roche, France) with 50 µL samples collected from the tail vein. Animals with glucose levels exceeding 300 mg/dL were considered diabetic, and neuropathic pain sensitivity assays were conducted 24 h later (three weeks following STZ injection).

Effect on the Mechanical Nociceptive Threshold (Von Frey Test)

The antiallodynic effect of compound **10** in STZ-induced neuropathic mice was evaluated using the von Frey test. Measurements were carried out with an electronic von Frey device (Bioseb, Vitrolles, France) equipped with a flexible filament that applied a gradually increasing force (0–10 g) to the plantar surface of the hind paw. Paw withdrawal terminated the stimulus, and the force triggering the response was automatically recorded. On day 21 after STZ administration, animals were placed individually in wire mesh-bottom chambers and habituated for 1 h. Baseline (predrug) sensitivity was then determined by testing each hind paw three times in alternation, with at least 30 s intervals between measurements. Then, the mice were pretreated with **10** or vehicle, and 60 min later, the animals were tested again to obtain postdrug values of neuropathic pain sensitivity [47].

Effect on the Heat Nociceptive Threshold (Hot Plate Test)

The hot plate assay was conducted in STZ-treated mice immediately after the von Frey test. The apparatus (Hot/Cold Plate, Bioseb, Vitrolles, France) consists of an electrically heated surface with a temperature control set to 55–56 °C. The latency to a nocifensive reaction (hind paw licking or jumping) was measured with a stopwatch. To prevent tissue injury, a cut-off time of 60 s was applied; animals not responding within this period were removed and assigned a latency of 60 s. Baseline (predrug) responses were determined before treatment, and postdrug latencies were measured 60 min after compound administration, at the same time point as the von Frey assessment [47].

Effect on Pancreatic Cell Architecture and Sciatic Nerve Structure

The isolated mouse tissues (pancreases and sciatic nerves) from each experimental group were fixed in 10% buffered formalin. After 24 h, sections were cut and processed in an Excelsior AS automatic vacuum processor from Epredia. The sections were then embedded in paraffin blocks using a MYR EC500 embedding station and cut into slides using a Leica Histocore Autocut automatic rotary microtome to a thickness of 2.5 µm. The sections were stained in the Sakura Tissue-Tek Prisma automatic staining system using the Sakura H&E staining kit. After staining, the slides were observed under a Nikon Eclipse E800 brightfield microscope. The morphological changes observed in the sections of STZ-Veh and STZ-compd**10**-exposed mouse tissues were examined, photographed with Canon EOS 80D, and compared with the control.

#### 3.6.4. Data Analysis

Data from the in vivo experiments were analyzed with GraphPad Prism 9.0. Results are presented as mean ± SEM. Statistical comparisons were performed using repeated-measures ANOVA with Dunnett’s or Sidak’s post hoc tests, or one-way ANOVA with Dunnett’s test. A *p* value < 0.05 was considered statistically significant. The Mann–Whitney U test was performed using PAST software, version 5.2.2, developed by Prof. Øyvind Hammer from the Natural History Museum, University of Oslo [50].

## 4. Conclusions

In this study, we designed and synthesized a novel series of 1-(2-biphenyl)piperazine derivatives with a hydantoin core to achieve dual-target activity at the 5-HT_7_R and voltage-gated sodium channels. Among them, compounds **10** and **20** exhibited high affinity for 5-HT_7_R (K_i_ < 10 nM) and potent sodium channel inhibition at 1 µM (>80%). Molecular docking indicated binding poses consistent with known 5-HT_7_R ligands. Cytotoxicity assays demonstrated acceptable hepatocyte and neuronal safety profiles, particularly for compound **10**, which showed higher IC_50_ values in both HepG2 and SH-SY5Y cells compared to compound **20**. In vitro metabolism studies revealed slightly improved microsomal stability compared with verapamil and relatively low potential for CYP-mediated DDIs at pharmacologically relevant concentrations. In vivo, compound **10** displayed significant antidepressant- and anxiolytic-like activity in behavioral models (FST and FPT), supporting its potential role in CNS modulation through 5-HT_7_ receptor interaction. However, despite its sodium channel blocking activity, compound **10** did not demonstrate efficacy in the STZ-induced diabetic neuropathy model. Overall, compound **10** appears to be a promising candidate for mood disorder therapy and warrants further in-depth investigation to support its future development.

## Figures and Tables

**Table 1:** Structures of compounds **9**–**22** with 5-HT_7_R binding affinity and % inhibition of sodium channel (site 2).

Compd	R_1_/R_2_ 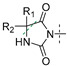	*n*	R_3_/R_4_	5-HT_7_ K_i_ [nM] ± SD	Na^+^ Channel (Site 2): % Inhibition of Control Specific Binding 100/10/1 [µM] *
**9**	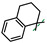	5	F/H	6 ± 1	55.6/-/-
**10**	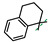	4	F/H	8 ± 2	107.2/99.3/88.6
**11**	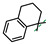	3	F/H	46 ± 11	83.6/-/-
**12**	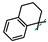	4	H/H	14 ± 2	-/-/-
**13**	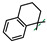	4	F/F	81 ± 18	-/-/-
**14**	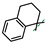	4	F/OCH_3_	143 ± 23	-/-/-
**15**	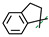	4	F/H	17 ± 3	-/-/-
**16**	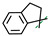	4	H/H	12 ± 2	-/-/-
**17**	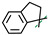	4	F/F	46 ± 11	-/-/-
**18**	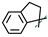	4	F/OCH_3_	47 ± 7	-/-/-
**19**	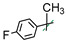	4	F/H	34 ± 5	-/-/-
**20**	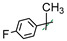	4	H/H	5 ± 1	-/97.8/83.0
**21**	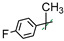	4	F/F	131 ± 12	-/-/-
**22**	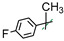	4	F/OCH_3_	65 ± 4	-/-/-
LP-211 **	-	-	-	15 ± 1	-/-/-
PHE ***	-	-	-	-	53.9/-/-

* Activities > 50% were considered significant, 25–50% as weak, and <25% as negligible. Binding studies were performed by Eurofins Laboratories (France), and all experiments were performed in duplicate; - not tested, ** data from Hedlund et al. [30], *** PHE—phenytoin, the reference drug tested under the same conditions.

**Table 2:** The metabolic stability and metabolic pathways of compound **10**. The main metabolic pathways were marked in bold.

Compd/Drug	Molecular Mass (*m/z*)	% Remaining	Molecular Mass of the Metabolite (*m/z*)	Metabolic Pathway
**10**	527.31	50.18	**543.33 (M1)** 257.18 (M2) 543.33 (M3) 559.30 (M4) 273.15 (M5)	**hydroxylation** decomposition hydroxylation dihydroxylation decomposition
**Verapamil**	455.31	30.80%	**441.35 (M1)** 291.32 (M2) 165.09 (M3) 441.29 (M4) 427.33 (M5) 277.26 (M6)	**demethylation** defragmentation defragmentation demethylation didemethylation defragmentation

## Data Availability

Compound samples are available from the corresponding author upon reasonable request.

## Supplementary Materials

The following supporting information can be downloaded at: https://www.mdpi.com/article/10.3390/ph18101485/s1, Figure S1: Cytotoxic effect of tested compounds in HepG2 cells; Figure S2: Cytotoxic effect of tested compounds in SH-SY5Y cells; Figure S3: LC-MS spectra of the reaction mixture of (A) compound **10** incubated with HLMs for 120 min, (B) compound **10** remained after incubation with HLMs for 120 min, (C) MS spectra of compound **10** metabolites; Figure S4: The MetaSite 6.0.1. software prediction of (A) the most probable sites of compound **10** metabolism; (B) the most probable hydroxylations of compound **10**; Table S1: HepG2 and SH-SY5Y cells viability in the presence of **9**–**22** compounds; and ^1^H NMR and ^13^ CNMR spectra of final compounds (**9**–**22**).
