# Supplementary material for: Novel Dual 5-HT7 Antagonists and Sodium Channel Inhibitors as Potential Therapeutic Agents with Antidepressant and Anxiolytic Activities
Source: Pharmaceuticals (Basel). 2025 Oct 2;18(10):1485. doi: 10.3390/ph18101485 (PMC12566688; doi:10.3390/ph18101485)
Supplement: Supplementary file 1 [file pharmaceuticals-18-01485-s001.zip › pharmaceuticals-3827983-supplementary.pdf]

Supplementary Materials:

Novel Dual 5-HT7 Antagonists and Sodium Channel Inhibitors as Potential Therapeutic Agents with Antidepressant and Anxiolytic Activities

Anna Czopek [1,*], Paulina Koczurkiewicz-Adamczyk [2], Katarzyna Wójcik-Pszczoła [2], Daria Kornas [1], Wojciech Sitko [1], Adam Bucki [1], Michał Sapa [1], Krzysztof Kamiński [1], Grzegorz Satała [3], Beata Duszyńska [4],
Andrzej J. Bojarski [3], Gniewomir Latacz [5], Jacek Czopek [6], Joanna Szpor [6], Pola Dryja [7] and Kinga Sałat [7]

[1] Department of Medicinal Chemistry, Faculty of Pharmacy, Jagiellonian University Medical College,
9 Medyczna St., 30-688 Kraków, Poland; dariak29@op.pl (D.K.); wojto42@gmail.com (W.S.); adam.bucki@uj.edu.pl (A.B.); michal.piotr.sapa@doctoral.uj.edu.pl (M.S.); k.kaminski@uj.edu.pl (K.K.)
[2] Department of Pharmaceutical Biochemistry, Faculty of Pharmacy, Jagiellonian University Medical College, 9 Medyczna St., 30-688 Krakow, Poland; paulina.koczurkiewicz@uj.edu.pl (P.K.-A.); katarzynaanna.wojcik@uj.edu.pl (K.W.-P.)
[3] Department of Medicinal Chemistry, Maj Institute of Pharmacology, Polish Academy of Sciences,
12 Smętna St., 31-343 Kraków, Poland; satala@if-pan.krakow.pl (G.S.); bojarski@if-pan.krakow.pl (A.J.B.)
[4] Department of Phytochemistry, Maj Institute of Pharmacology, Polish Academy of Sciences, 12 Smętna St., 31-343 Kraków, Poland; duszyn@if-pan.krakow.pl
[5] Department of Technology and Biotechnology of Drugs, Faculty of Pharmacy, Jagiellonian University
Medical College, 9 Medyczna St., 30-688 Kraków, Poland; gniewomir.latacz@uj.edu.pl
[6] Department of Pathomorphology, Jagiellonian University Medical College,16 Grzegórzecka St.,
31-008 Kraków, Poland; jacek.czopek@uj.edu.pl (J.C.); joanna.szpor@uj.edu.pl (J.S.)
[7] Department of Pharmacodynamics, Chair of Pharmacodynamics, Faculty of Pharmacy, Jagiellonian
University Medical College, 9 Medyczna St., 30-688 Kraków, Poland; pola.dryja@student.uj.edu.pl (P.D.); kinga.salat@uj.edu.pl (K.S.)
* Correspondence: anna.czopek@uj.edu.pl; Tex.: +48-12-6205450

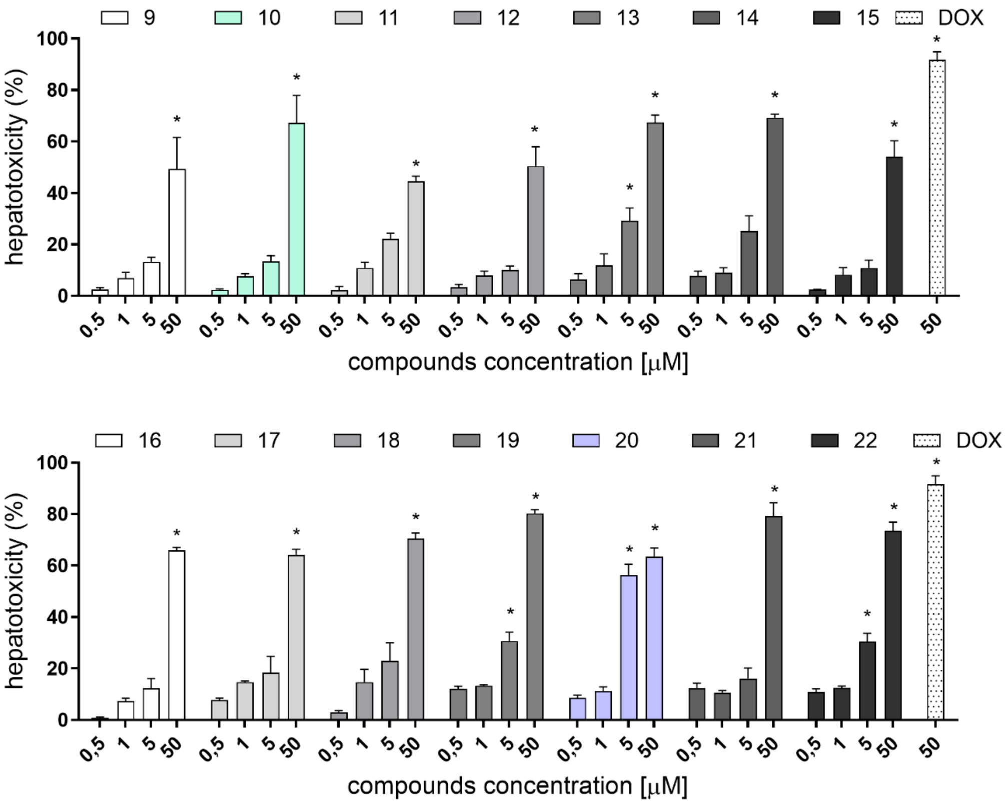

Figure S1. Cytotoxic effect of tested compounds in HepG2 cells.

Cells were seeded at a density of $5*10^3$ cells/well and after overnight culture exposed to growing concentrations (0.5-100 μM) of tested compounds for 24 h. Hepatocytotoxicity was measured by CyQUANT™ LDH Cytotoxicity Assay Kit assessing LDH release into the culture medium as an indicator of plasma membrane integrity. Bars represents mean ± SEM of percent cytotoxicity obtained in three independent experiments. Results were compared to positive control – doxorubicin (DOX). * Statistical significance versus control ($p < 0.05$).

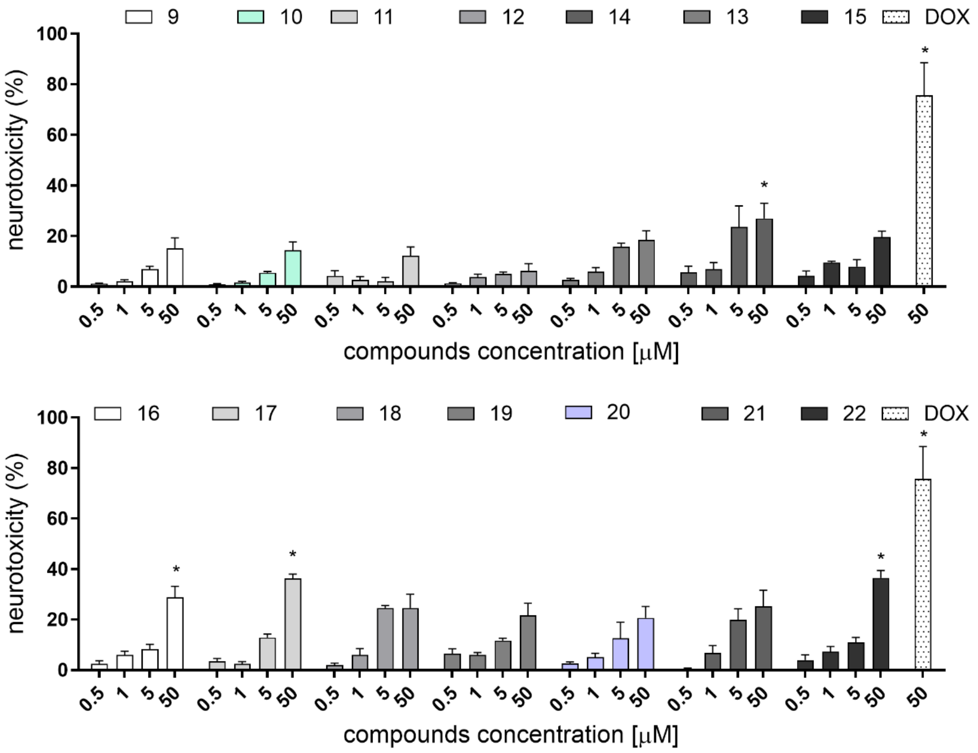

Figure S2. Cytotoxic effect of tested compounds in SH-SY5Y cells.

Cells were seeded at a density of 5*10^3 cells/well and after overnight culture exposed to growing concentrations (0.5-100 µM) of tested compounds for 24 h. Neurocytotoxicity was measured by CyQUANT™ LDH Cytotoxicity Assay Kit assessing LDH release into the culture medium as an indicator of plasma membrane integrity. Bars represents mean ± SEM of percent cytotoxicity obtained in three independent experiments. Results were compared to positive control – doxorubicin (DOX). * Statistical significance versus control ($p < 0.05$).

Table S1. HepG2 and SH-SY5Y cells viability in the presence of **9-22** compounds**.**

Cmpd	HepG2 IC_{50} [µM]	SH-SY5Y IC_{50} [µM]
9	18.06	25.99
10	7.55	24.63
11	32.36	74.01
12	12.32	42.15
13	7.5	10.44
14	9.44	12.43
15	22.38	27.31
16	5.68	12.03
17	12.66	28.02
18	10.82	9.75
19	3.45	14.34
20	3.15	17.23
21	5.79	10.26
22	4.46	13.83
DOX	2.98	3.37

Cells were seeded at a density of 5*10^3 cells/well and after overnight culture exposed to growing concentrations (0.5-100 µM) of tested compounds for 24 h. Cells viability was assessed using the MTT assay. IC_{50} values were calculated using nonlinear regression analysis.

A

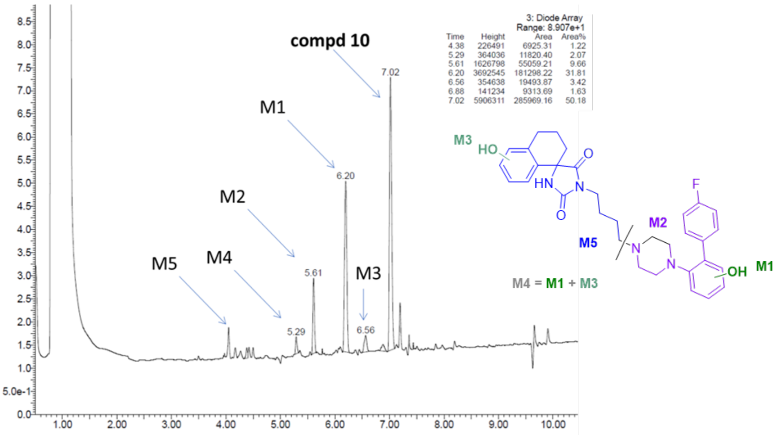

B

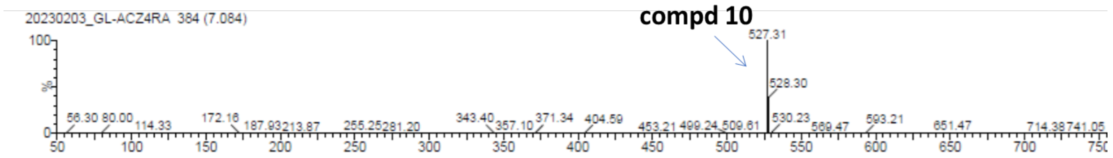

C

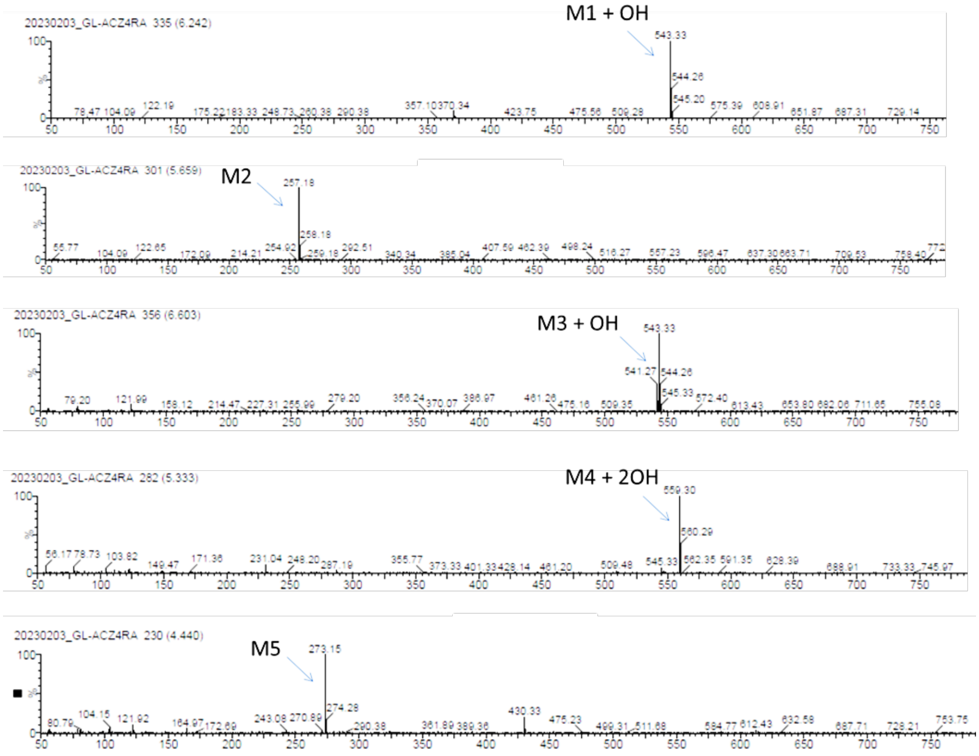

Figure S3. LC-MS spectra of the reaction mixture of (A) compound **10** incubated with HLMs for 120 min. (B) compound **10** remained after incubation with HLMs for 120 min. (C) MS spectra of compound **10** metabolites.

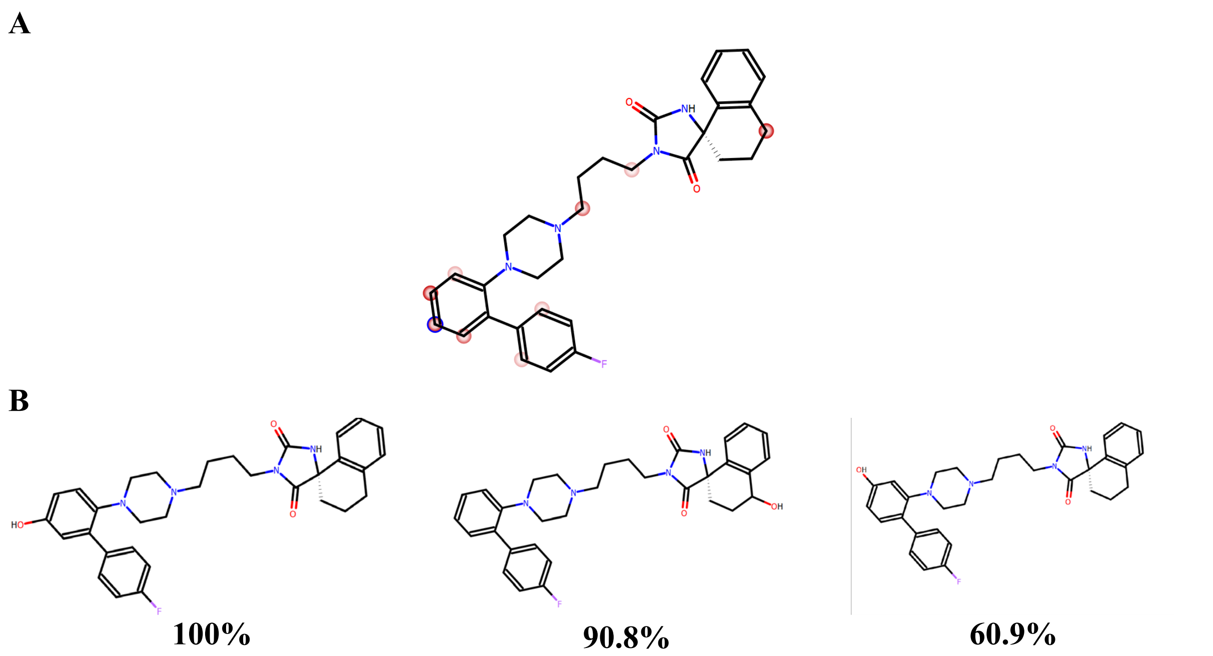

Figure S4. The MetaSite 6.0.1. software prediction of (A) the most probable sites of compound **10** metabolism; (B) the most probable hydroxylations of compound **10**. The darker red color - the higher probability of being involved in the metabolism pathway.

^{1}H NMR and ^{13} CNMR spectra of final compounds (**9**-**22**)

Compd 9

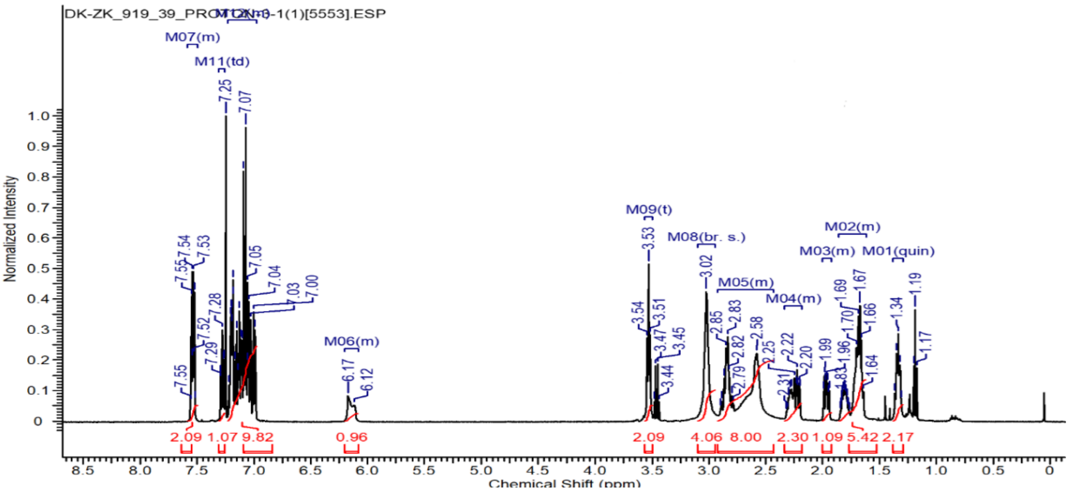

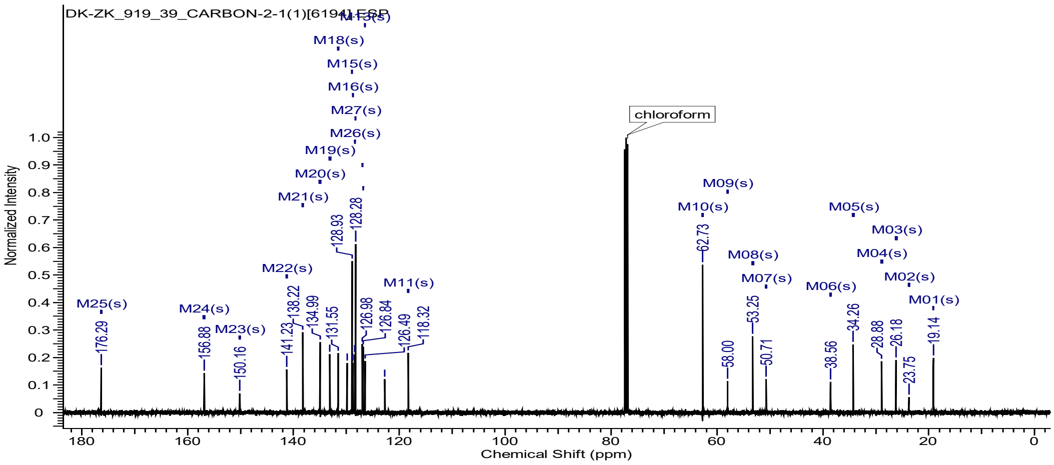

Compd 10

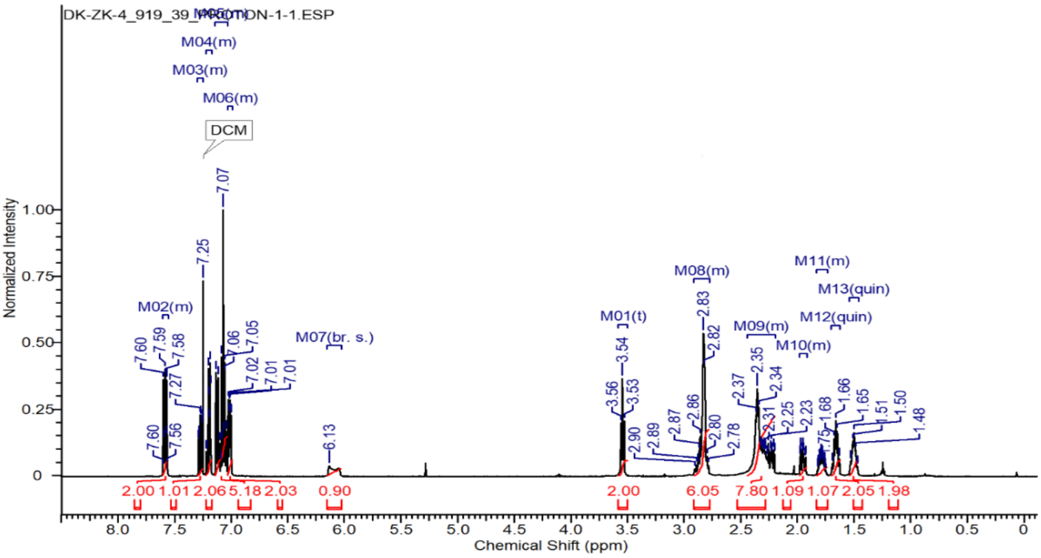

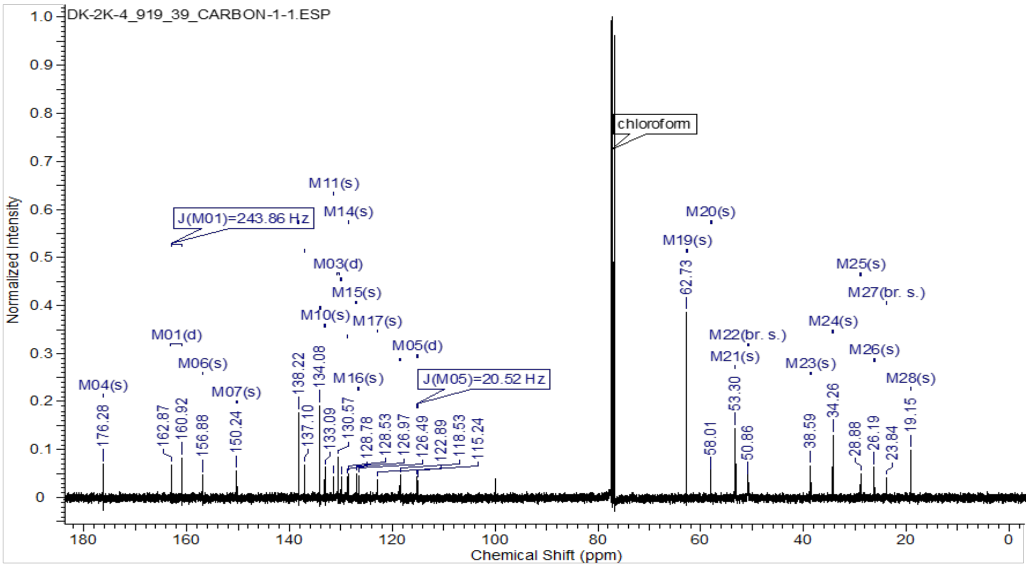
DK-2K-4_919_39_CARBON-1-1.ESP
Normalized Intensity
Chemical Shift (ppm)
chloroform
J(M01)=243.86 Hz
J(M05)=20.52 Hz
M04(s) M01(d) M06(s) M07(s) M11(s) M14(s) M03(d) M15(s) M10(s) M17(s) M05(d) M16(s) M20(s) M19(s) M22(br. s.) M21(s) M23(s) M24(s) M25(s) M27(br. s.) M26(s) M28(s)
176.28 162.87 160.92 156.88 150.24 138.22 137.10 134.08 133.09 130.57 128.78 128.53 126.97 126.49 122.89 118.53 115.24
62.73 58.01 53.30 50.86 38.59 34.26 28.88 26.19 23.84 19.15

Compd 11

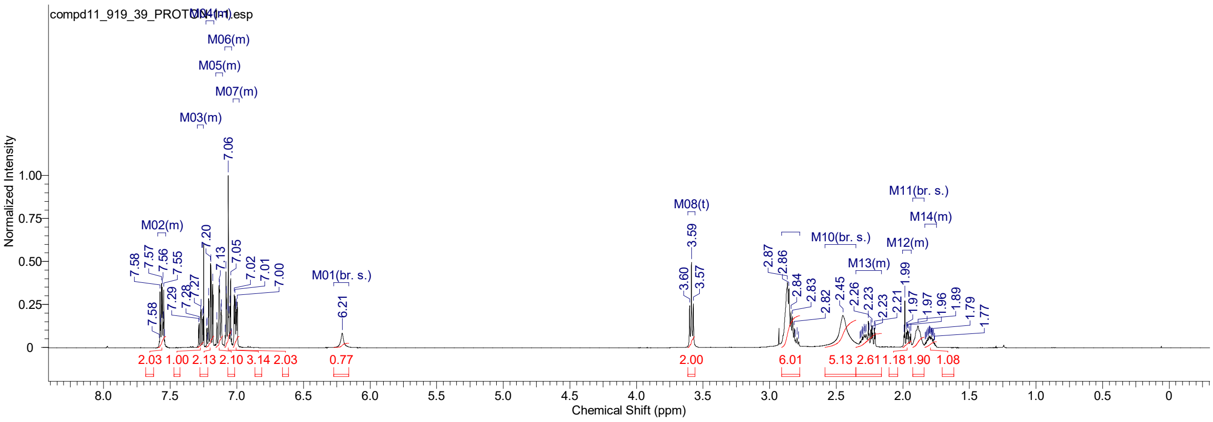
compd11_919_39_PROTON-1-1.esp
Normalized Intensity
Chemical Shift (ppm)
M02(m) M03(m) M04(m) M05(m) M06(m) M07(m) M01(br. s.) M08(t) M10(br. s.) M11(br. s.) M12(m) M13(m) M14(m)
7.58 7.57 7.56 7.55 7.58 7.29 7.28 7.27 7.20 7.13 7.06 7.05 7.02 7.01 7.00 6.21 3.60 3.59 3.57
2.87 2.86 2.84 2.83 2.82 2.45 2.26 2.23 2.23 2.21 1.99 1.97 1.97 1.96 1.89 1.79 1.77
2.03 1.00 2.13 2.10 3.14 2.03 0.77 2.00 6.01 5.13 2.61 1.18 1.90 1.08

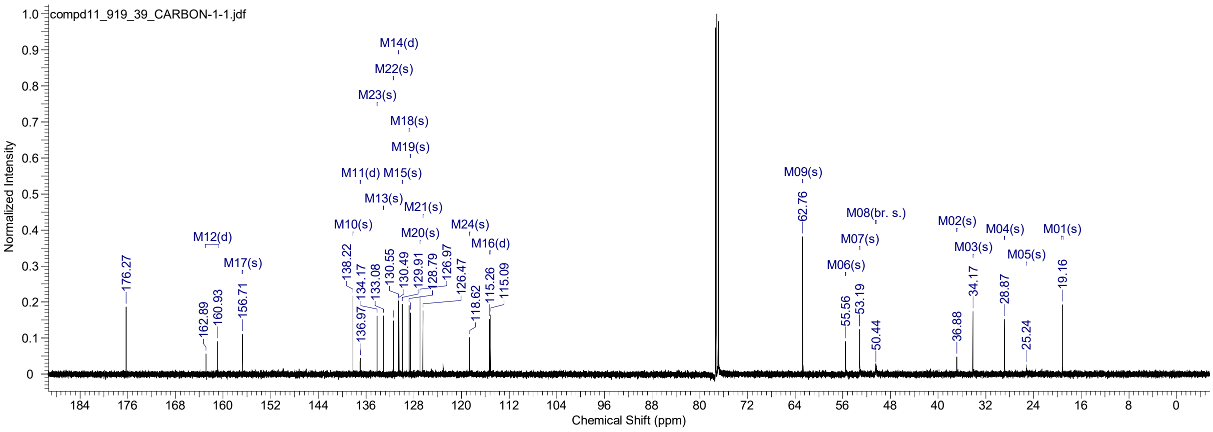
compd11_919_39_CARBON-1-1.jdf
Normalized Intensity
Chemical Shift (ppm)
M14(d) M22(s) M23(s) M18(s) M19(s) M11(d) M15(s) M13(s) M21(s) M10(s) M20(s) M24(s) M16(d) M12(d) M17(s) M09(s) M08(br. s.) M07(s) M06(s) M02(s) M04(s) M01(s) M03(s) M05(s)
176.27 162.89 160.93 156.71 138.22 136.97 134.17 133.08 130.55 130.49 129.91 128.79 126.97 126.47 118.62 115.26 115.09
62.76 55.56 53.19 50.44 36.88 34.17 28.87 25.24 19.16

Compd 12

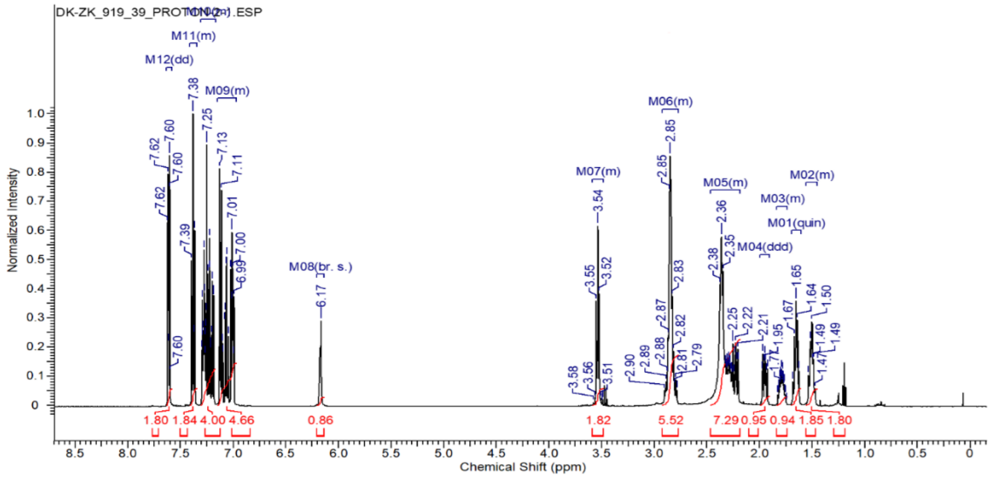

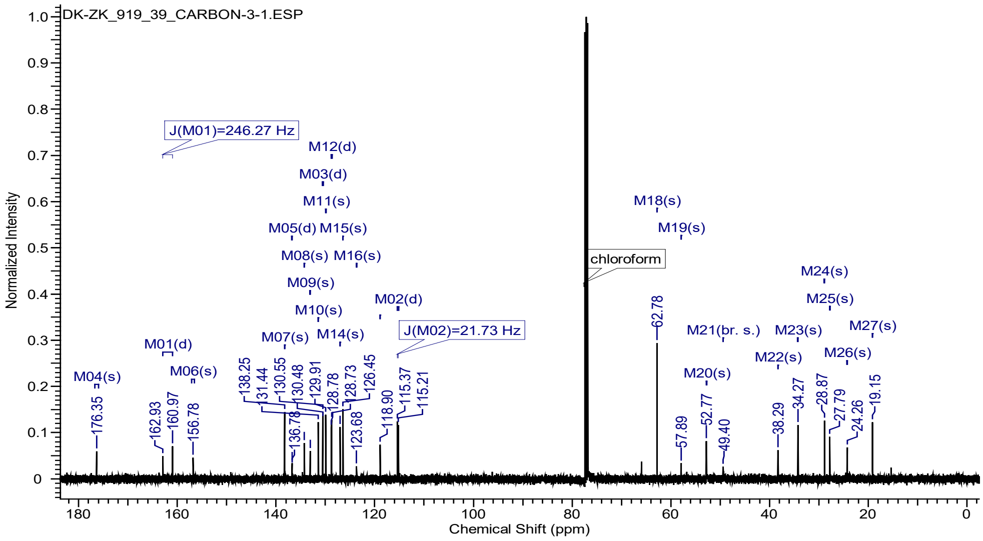

Compd 13

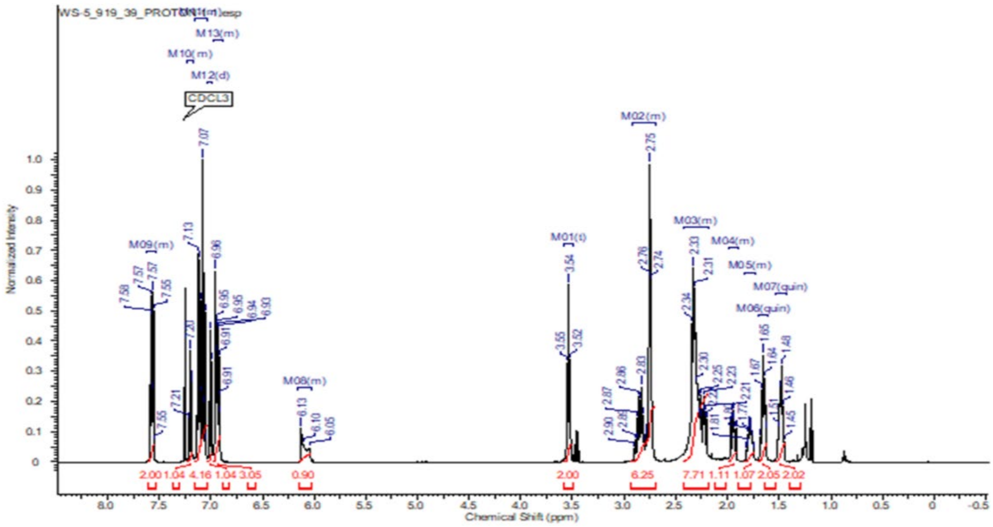

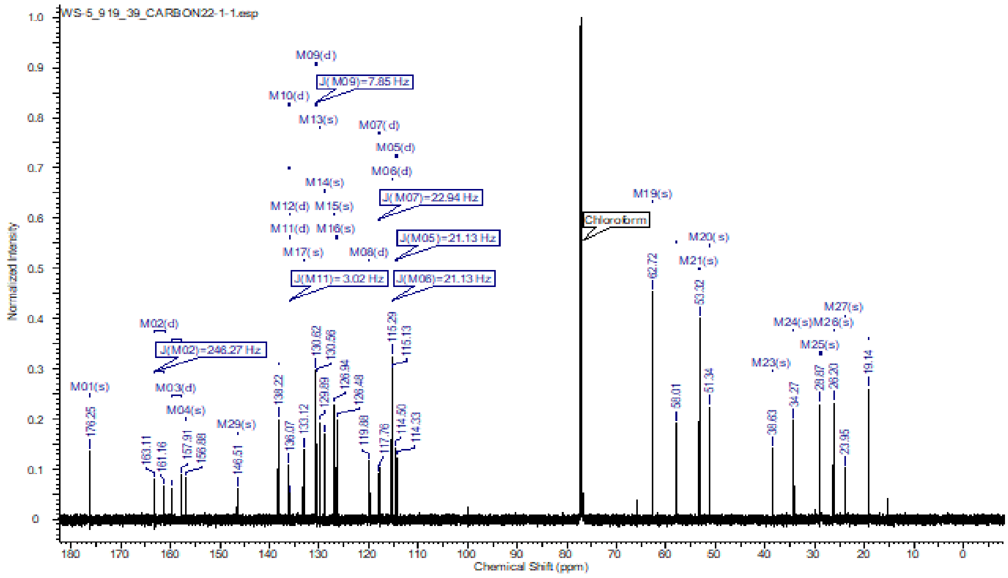
WS-5_919_39_CARBON22-1-1.esp
Normalized Intensity
Chemical Shift (ppm)
Chloroform
M01(s)
M02(d)
J(M02)=246.27 Hz
M03(d)
M04(s)
M05(d)
J(M05)=21.13 Hz
M06(d)
J(M06)=21.13 Hz
M07(d)
J(M07)=22.94 Hz
M08(d)
M09(d)
J(M09)=7.85 Hz
M10(d)
M11(d)
J(M11)=3.02 Hz
M12(d)
M13(s)
M14(s)
M15(s)
M16(s)
M17(s)
M19(s)
M20(s)
M21(s)
M23(s)
M24(s)
M25(s)
M26(s)
M27(s)
M29(s)
176.25
163.11
161.16
157.91
156.88
146.51
138.22
136.07
133.12
130.62
130.58
129.89
128.94
128.48
119.08
117.76
115.29
115.13
114.50
114.33
62.72
58.01
53.32
51.34
38.63
34.27
28.87
26.20
23.95
19.14

Compd 14

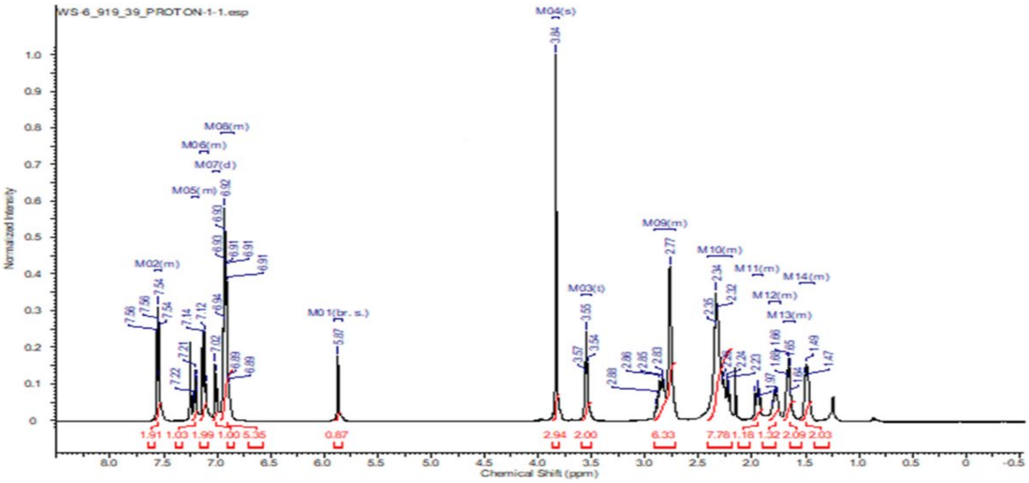
WS-6_919_39_PROTON-1-1.esp
Normalized Intensity
Chemical Shift (ppm)
M01(br. s.)
M02(m)
M03(t)
M04(s)
M05(m)
M06(m)
M07(d)
M08(m)
M09(m)
M10(m)
M11(m)
M12(m)
M13(m)
M14(m)
7.56
7.56
7.54
7.54
7.22
7.21
7.14
7.12
7.02
6.94
6.93
6.92
6.91
6.91
6.89
6.89
5.87
3.84
3.57
3.55
3.54
2.88
2.86
2.85
2.83
2.77
2.35
2.34
2.32
2.24
2.23
1.97
1.68
1.66
1.65
1.64
1.49
1.47
1.91
1.03
1.99
1.00
5.35
0.87
2.94
2.00
6.33
7.78
1.18
1.32
2.09
2.03

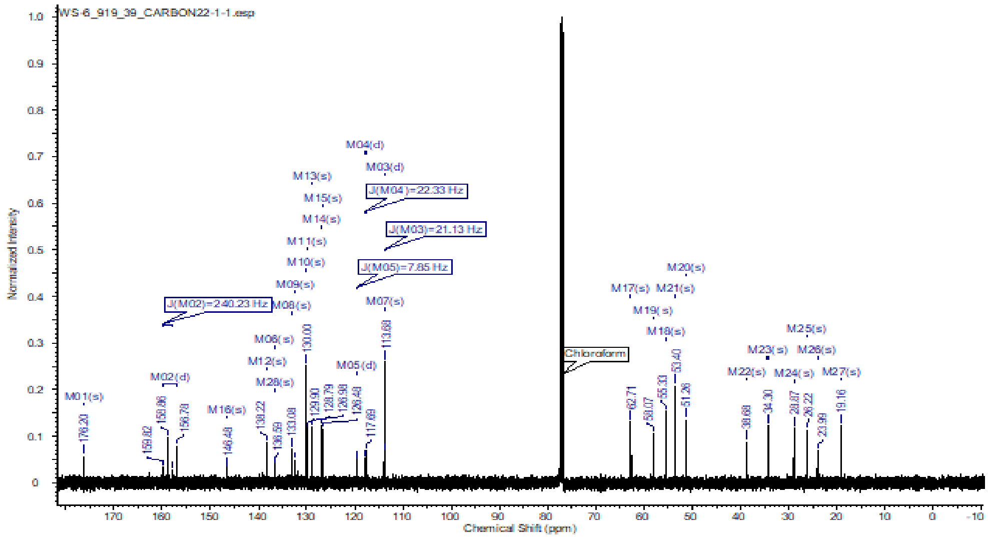
WS-6_919_39_CARBON22-1-1.esp
Normalized Intensity
Chemical Shift (ppm)
Chloroform
M01(s)
M02(d)
J(M02)=240.23 Hz
M03(d)
J(M03)=21.13 Hz
M04(d)
J(M04)=22.33 Hz
M05(d)
J(M05)=7.85 Hz
M06(s)
M07(s)
M08(s)
M09(s)
M10(s)
M11(s)
M12(s)
M13(s)
M14(s)
M15(s)
M16(s)
M17(s)
M18(s)
M19(s)
M20(s)
M21(s)
M22(s)
M23(s)
M24(s)
M25(s)
M26(s)
M27(s)
M28(s)
176.20
159.82
158.86
156.78
146.48
138.22
136.59
133.08
130.00
129.90
128.79
128.98
128.48
117.69
113.68
62.71
58.07
55.33
53.40
51.26
38.68
34.30
28.87
26.22
23.99
19.16

Compd 15

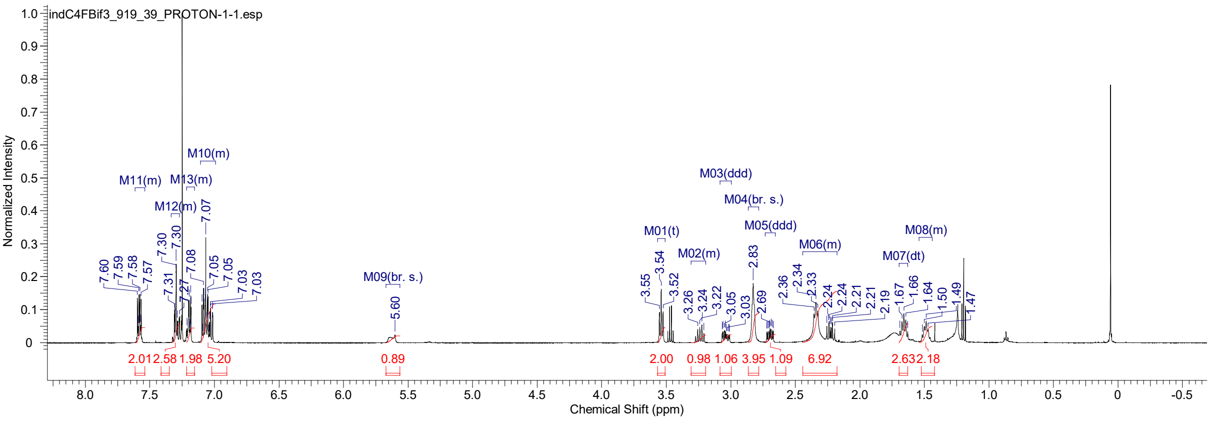

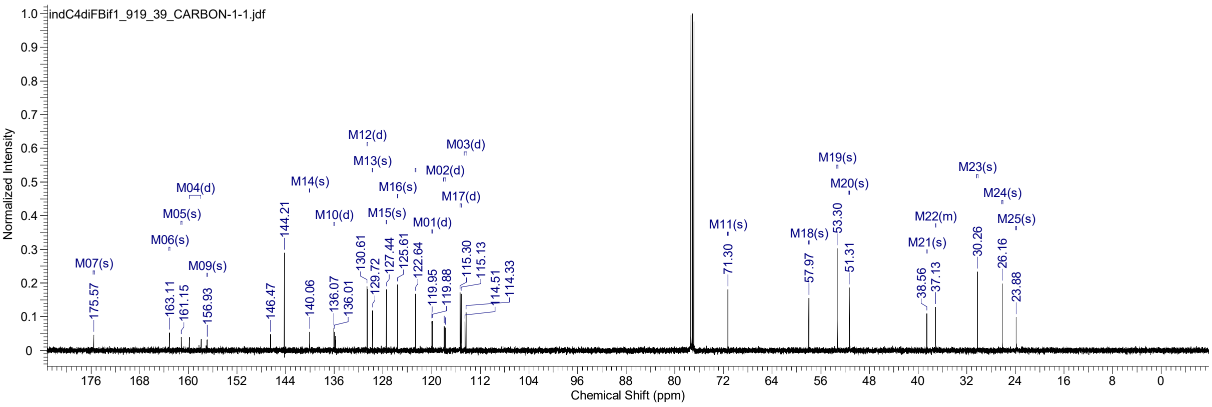

Compd 16

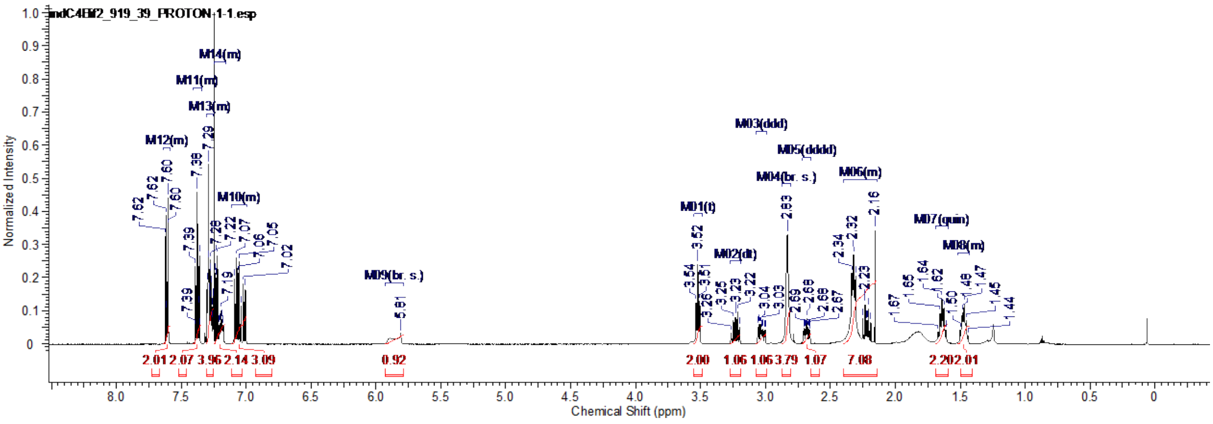

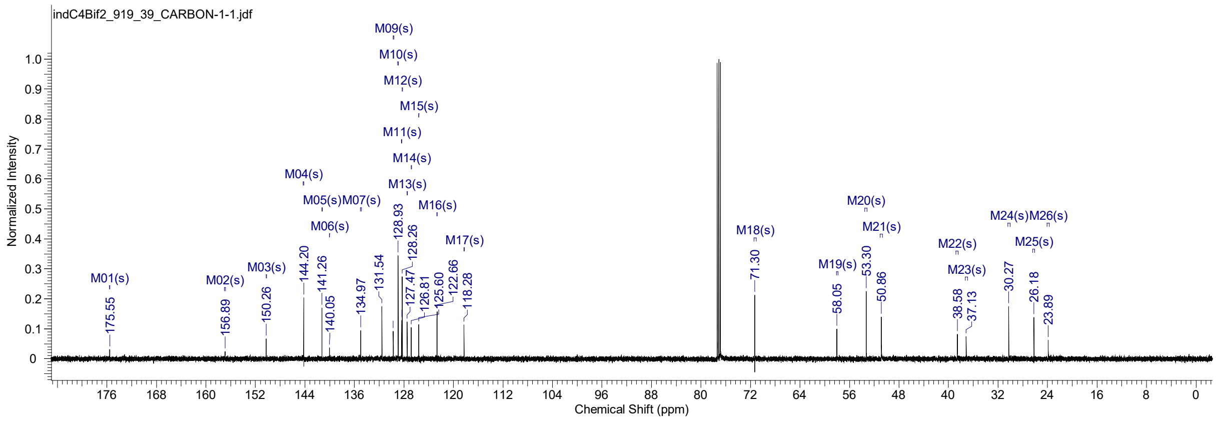

Compd 17

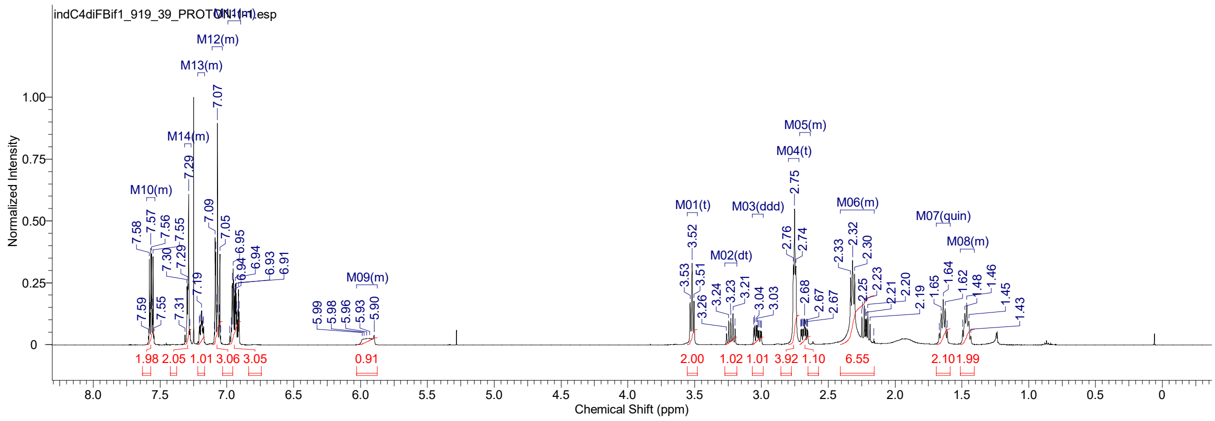

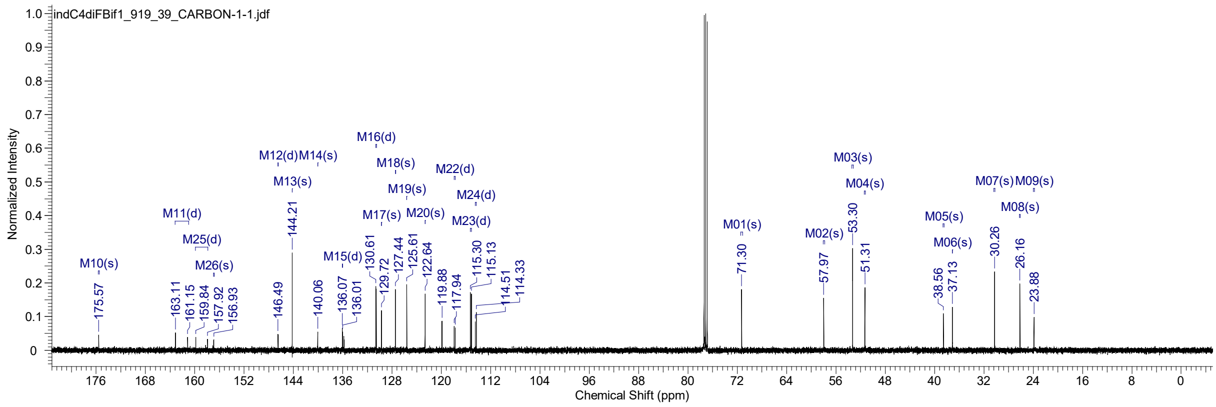

Compd 18

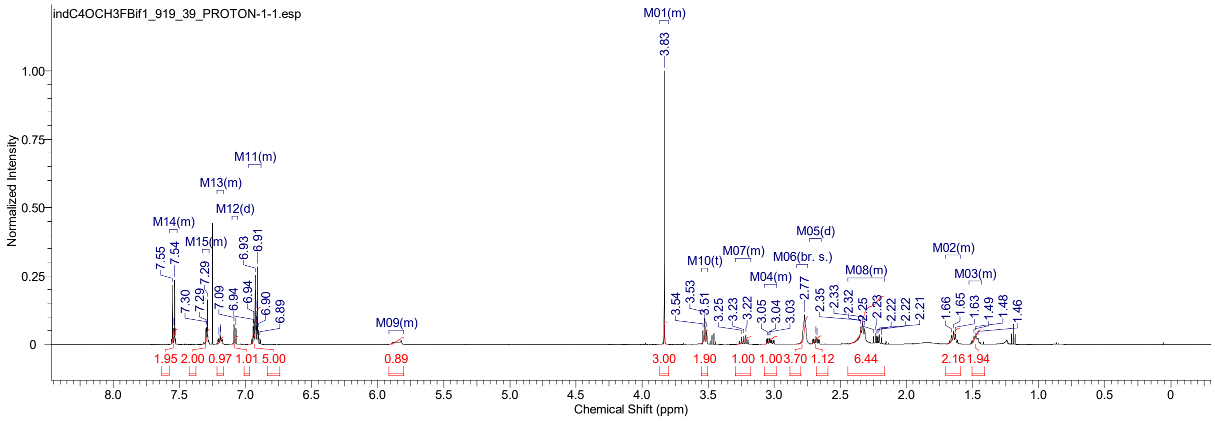

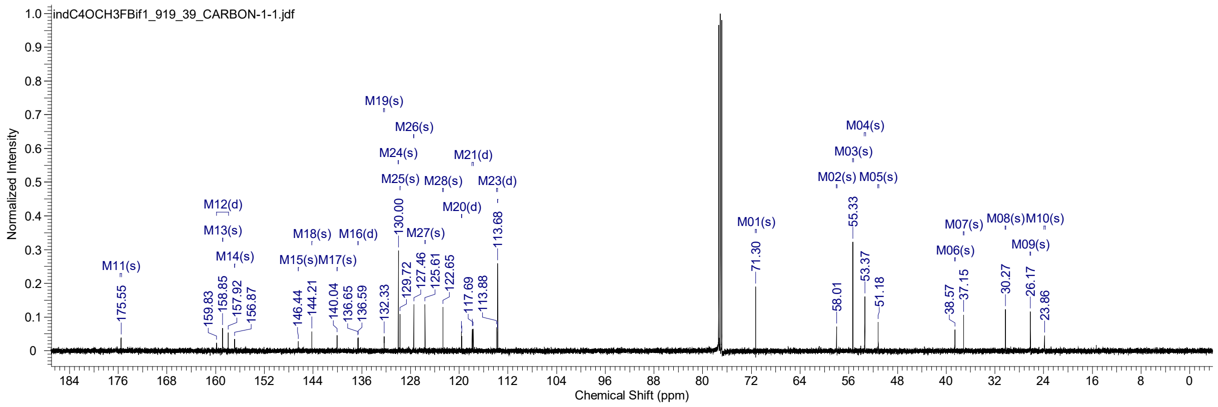
indC4OCH3FBif1_919_39_CARBON-1-1.jdf
Normalized Intensity
Chemical Shift (ppm)
175.55
159.83
158.85
157.92
156.87
146.44
144.21
140.04
136.65
136.59
132.33
130.00
129.72
127.46
125.61
122.65
117.69
113.88
113.68
71.30
58.01
55.33
53.37
51.18
38.57
37.15
30.27
26.17
23.86

Compd 19

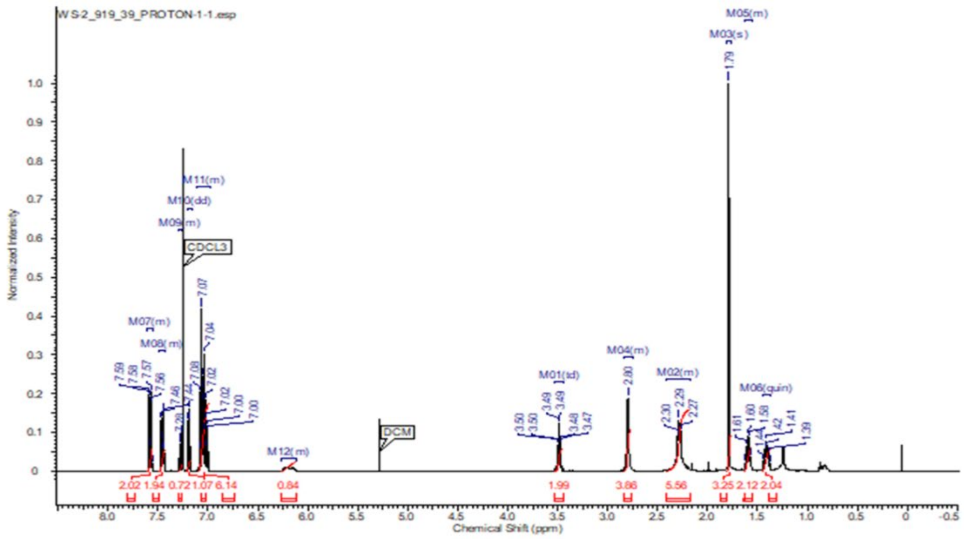
WS-2_919_39_PROTON-1-1.esp
Normalized Intensity
Chemical Shift (ppm)
CDCL3
DCM
2.02
1.94
0.72
1.07
6.14
0.84
1.99
3.86
5.56
3.25
2.12
2.04

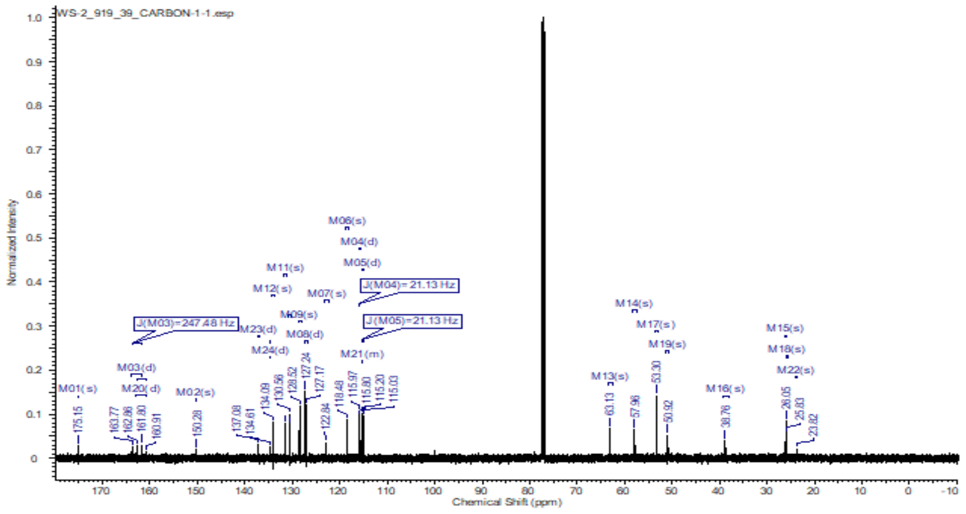
WS-2_919_39_CARBON-1-1.esp
Normalized Intensity
Chemical Shift (ppm)
J(M03)=247.48 Hz
J(M04)= 21.13 Hz
J(M05)=21.13 Hz
175.15
163.77
162.86
161.80
160.91
150.28
137.08
134.61
134.09
130.56
128.52
127.24
127.17
122.84
118.48
115.97
115.80
115.20
115.03
63.13
57.96
53.30
50.92
38.78
26.05
25.83
23.82

Compd 20

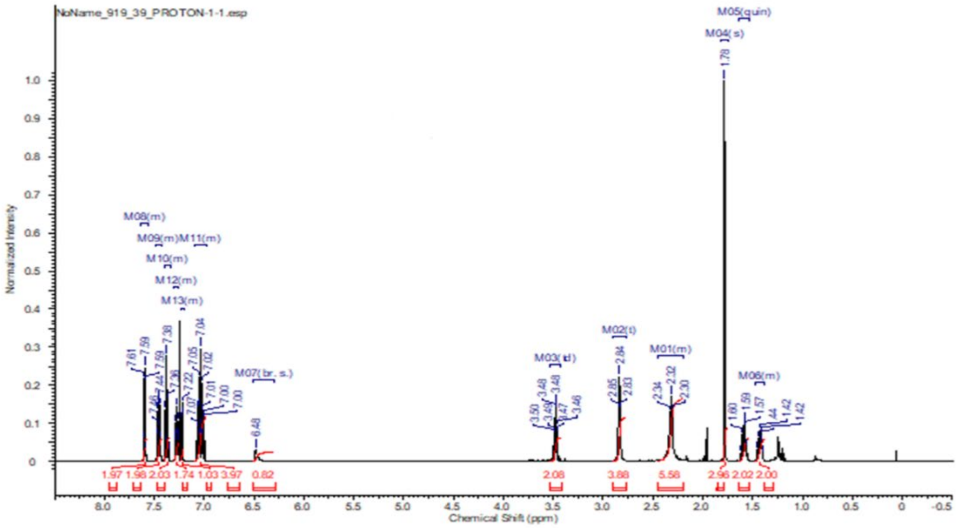

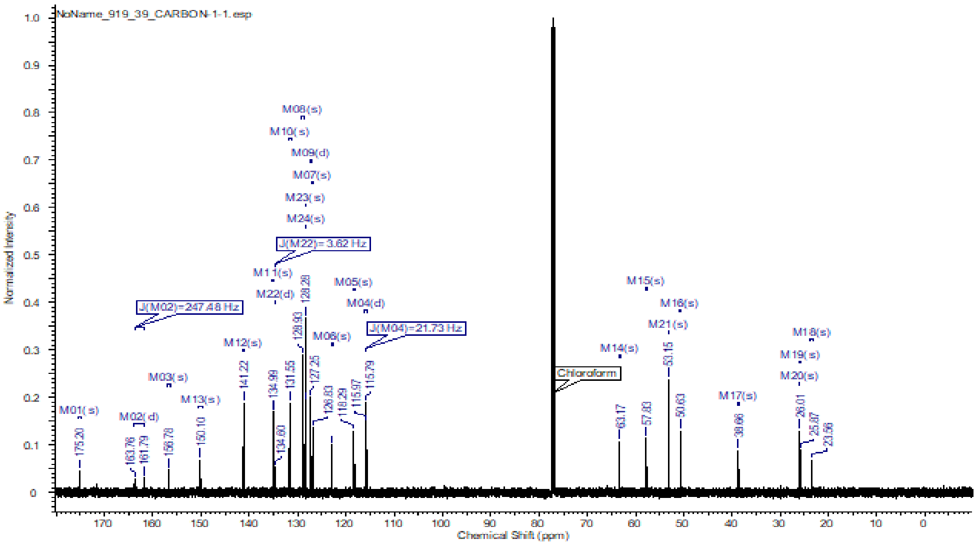

Compd 21

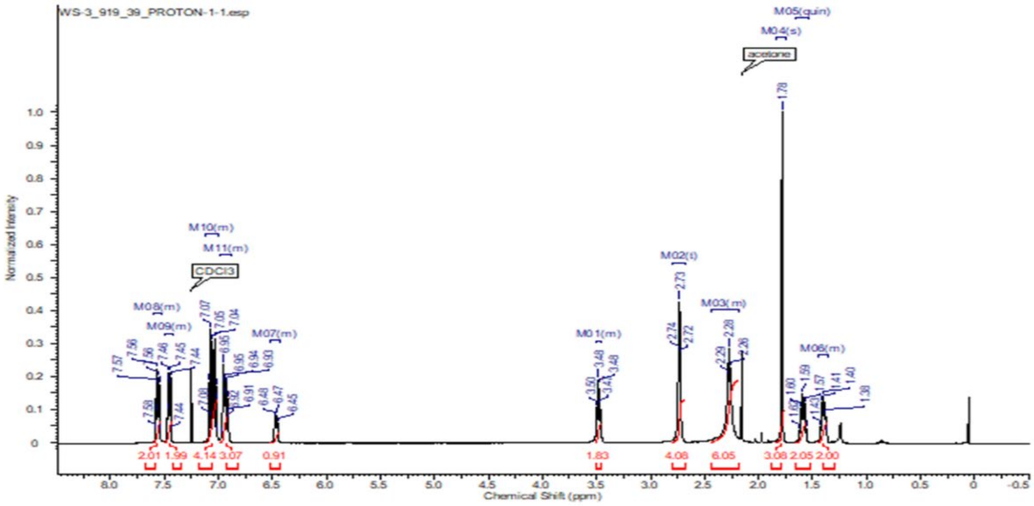

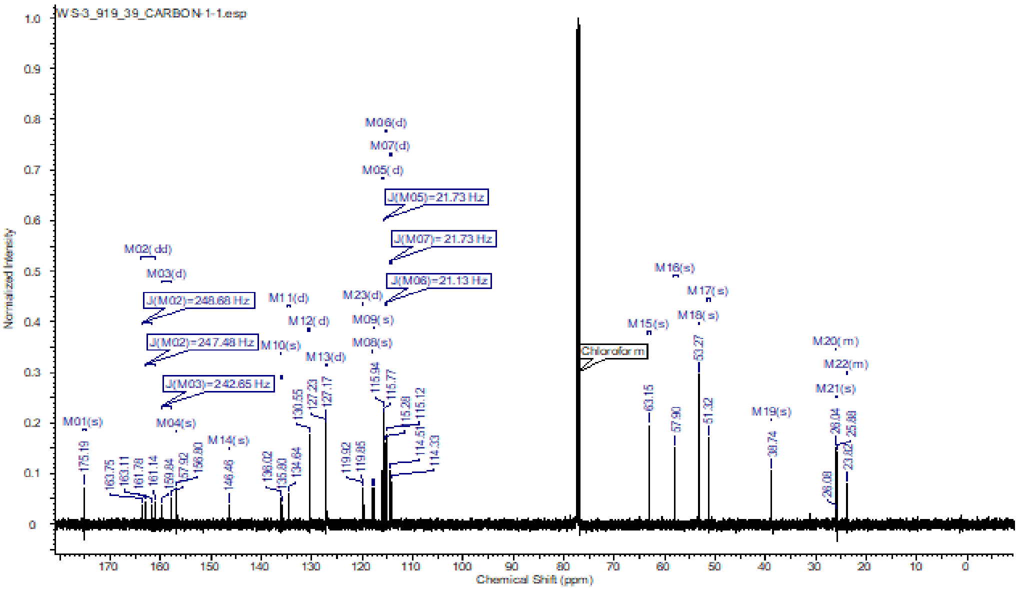
WS-3_919_39_CARBON-1-1.esp
Normalized Intensity
Chemical Shift (ppm)
Chloroform
M01(s)
M02(dd)
M03(d)
M04(s)
M05(d)
M06(d)
M07(d)
M08(s)
M09(s)
M10(s)
M11(d)
M12(d)
M13(d)
M14(s)
M15(s)
M16(s)
M17(s)
M18(s)
M19(s)
M20(m)
M21(s)
M22(m)
M23(d)
J(M05)=21.73 Hz
J(M07)=21.73 Hz
J(M06)=21.13 Hz
J(M02)=248.68 Hz
J(M02)=247.48 Hz
J(M03)=242.65 Hz
175.19
163.75
163.11
161.78
161.14
159.84
157.92
156.80
146.48
136.02
135.80
134.64
130.55
127.23
127.17
119.92
119.85
115.94
115.77
115.28
115.12
114.51
114.33
63.15
57.90
53.27
51.32
38.74
26.08
26.04
25.88
23.82

Compd 22

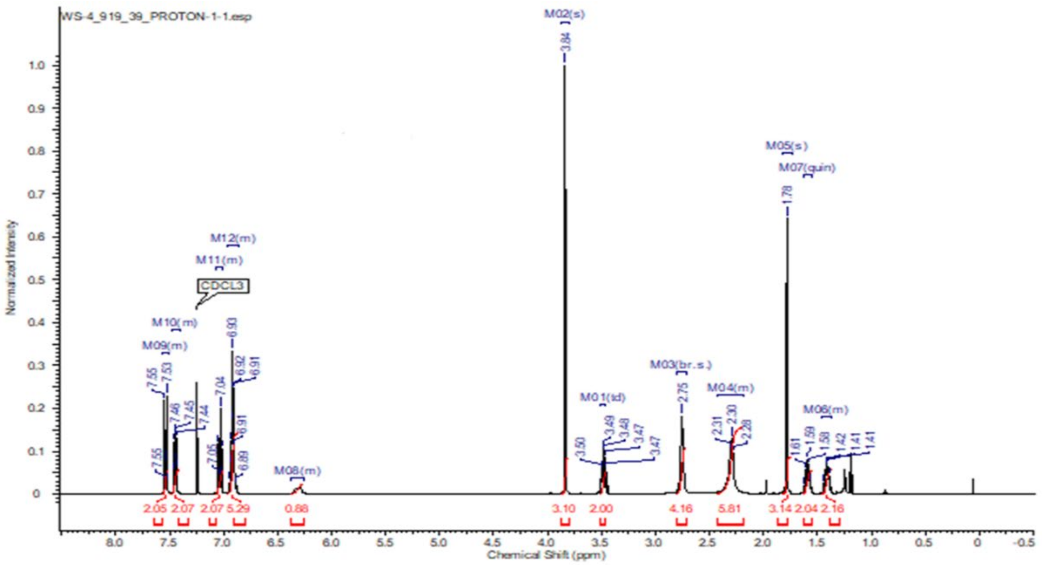
WS-4_919_39_PROTON-1-1.esp
Normalized Intensity
Chemical Shift (ppm)
CDCL3
M01(td)
M02(s)
M03(br. s.)
M04(m)
M05(s)
M06(m)
M07(quin)
M08(m)
M09(m)
M10(m)
M11(m)
M12(m)
3.84
1.78
7.55
7.53
7.46
7.45
7.44
7.04
7.05
6.93
6.92
6.91
6.89
3.50
3.49
3.48
3.47
2.75
2.31
2.30
2.28
1.61
1.59
1.58
1.42
1.41
2.05
2.07
2.07
5.29
0.88
3.10
2.00
4.16
5.81
3.14
2.04
2.16

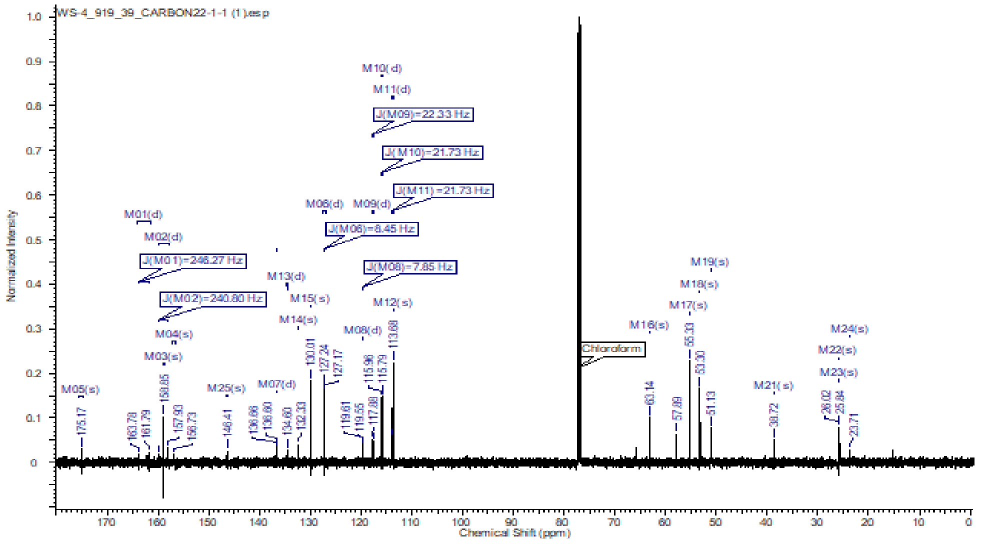
WS-4_919_39_CARBON22-1-1 (1).esp
Normalized Intensity
Chemical Shift (ppm)
Chloroform
M01(d)
M02(d)
M03(s)
M04(s)
M05(s)
M06(d)
M07(d)
M08(d)
M09(d)
M10(d)
M11(d)
M12(s)
M13(d)
M14(s)
M15(s)
M16(s)
M17(s)
M18(s)
M19(s)
M21(s)
M22(s)
M23(s)
M24(s)
M25(s)
J(M09)=22.33 Hz
J(M10)=21.73 Hz
J(M11)=21.73 Hz
J(M06)=8.45 Hz
J(M08)=7.85 Hz
J(M01)=246.27 Hz
J(M02)=240.80 Hz
175.17
163.78
161.79
158.85
157.93
156.73
146.41
136.66
136.60
134.60
132.33
130.01
127.24
127.17
119.61
119.55
117.08
115.96
115.79
113.68
63.14
57.09
55.33
53.30
51.13
38.72
26.02
25.84
23.71
